# Hybrid Lipid/Polymer Nanoparticles to Tackle the Cystic Fibrosis Mucus Barrier in siRNA Delivery to the Lungs: Does PEGylation Make the Difference?

**DOI:** 10.1021/acsami.1c14975

**Published:** 2022-02-02

**Authors:** Gemma Conte, Gabriella Costabile, Domizia Baldassi, Valeria Rondelli, Rosaria Bassi, Diego Colombo, Giulia Linardos, Ersilia V. Fiscarelli, Raffaella Sorrentino, Agnese Miro, Fabiana Quaglia, Paola Brocca, Ivana d’Angelo, Olivia M. Merkel, Francesca Ungaro

**Affiliations:** † Di.S.T.A.Bi.F., University of Campania Luigi Vanvitelli, Caserta 81100, Italy; ‡ Department of Pharmacy, University of Napoli Federico II, Napoli 80131, Italy; § Department of Pharmacy, Pharmaceutical Technology and Biopharmacy, Ludwig-Maximilians-Universität, München, Munich 81377, Germany; ∥ Department of Medical Biotechnologies and Translational Medicine, University of Milano, Segrate (MI) 20090, Italy; ⊥ Children’s Hospital Bambino Gesù IRCCS, Rome 00165, Italy; # Department of Molecular Medicine and Medical Biotechnologies, University of Napoli Federico II, Napoli 80131, Italy

**Keywords:** lung mucus, hybrid nanoparticles, siRNA delivery, cystic fibrosis, SAXS

## Abstract

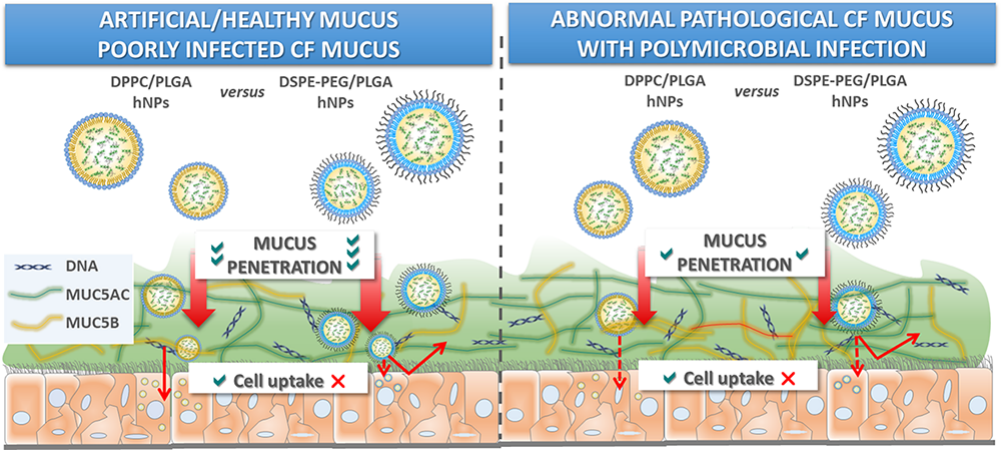

Inhaled siRNA therapy has a unique potential for treatment of severe lung diseases, such as cystic fibrosis (CF). Nevertheless, a drug delivery system tackling lung barriers is mandatory to enhance gene silencing efficacy in the airway epithelium. We recently demonstrated that lipid-polymer hybrid nanoparticles (hNPs), comprising a poly(lactic-co-glycolic) acid (PLGA) core and a lipid shell of dipalmitoyl phosphatidylcholine (DPPC), may assist the transport of the nucleic acid cargo through mucus-covered human airway epithelium. To study in depth the potential of hNPs for siRNA delivery to the lungs and to investigate the hypothesized benefit of PEGylation, here, an siRNA pool against the nuclear factor-κB (siNFκB) was encapsulated inside hNPs, endowed with a non-PEGylated (DPPC) or a PEGylated (1,2-distearoyl-*sn*-glycero-3-phosphoethanolamine-poly(ethylene glycol) or DSPE-PEG) lipid shell. Resulting hNPs were tested for their stability profiles and transport properties in artificial CF mucus, mucus collected from CF cells, and sputum samples from a heterogeneous and representative set of CF patients. Initial information on hNP properties governing their interaction with airway mucus was acquired by small-angle X-ray scattering (SAXS) studies in artificial and cellular CF mucus. The diffusion profiles of hNPs through CF sputa suggested a crucial role of lung colonization of the corresponding donor patient, affecting the mucin type and content of the sample. Noteworthy, PEGylation did not boost mucus penetration in complex and sticky samples, such as CF sputa from patients with polymicrobial colonization. In parallel, in vitro cell uptake studies performed on mucus-lined Calu-3 cells grown at the air–liquid interface (ALI) confirmed the improved ability of non-PEGylated hNPs to overcome mucus and cellular lung barriers. Furthermore, effective *in vitro* NFκB gene silencing was achieved in LPS-stimulated 16HBE14o- cells. Overall, the results highlight the potential of non-PEGylated hNPs as carriers for pulmonary delivery of siRNA for local treatment of CF lung disease. Furthermore, this study provides a detailed understanding of how distinct models may provide different information on nanoparticle interaction with the mucus barrier.

## Introduction

Lung delivery of small interfering RNA (siRNA) holds great promise for the treatment of severe lung diseases, such as cystic fibrosis (CF).^1,2^ As a matter of fact, synthetic therapeutic siRNA can be designed to virtually target any gene of interest with high selectivity, including those targets considered “undruggable”.^3^ Furthermore, the pulmonary route of administration offers the unprecedented opportunity to directly deliver siRNA to the diseased lung tissue in a loco-regional and minimally invasive manner.^1,4^ Nevertheless, despite the huge therapeutic potential, only two clinical trials providing for aerosolized siRNA have been undertaken till now (ALN-RSV-01, Alnylam Pharmaceuticals, Phase IIb completed; Excellair, ZaBeCor Pharmaceutical Co, Phase II discontinued), highlighting the intrinsic difficulties involved in the translation of inhaled siRNA from the bench to the clinics.

A key challenge to exploit the full potential of siRNA-based therapeutics for inhalation is the development of safe and effective delivery systems, assisting siRNA transport to their cell target at the lungs.^5^ An early investigated technological approach relies on lipid-polymer hybrid nanoparticles (hNPs) that is based on core–shell nanoparticles comprising a poly(lactic-co-glycolic) acid (PLGA) core and a lipid shell, exhibiting complementary characteristics of both polymeric nanoparticles and liposomes.^6−10^ Of note, we recently demonstrated that the lipid layer surrounding the PLGA core likely confers muco-inertia to hNPs, thus assisting the transport of the nucleic acid cargo through the mucus-covered human airway epithelial barrier.^11,12^

The mucus layer is composed of highly cross-linked mucin chains, water, and other gel-like constituents creating a complex barrier, which strongly affects the fate of any inhaled particle in the lungs.^13−15^ This is a crucial concern in severe lung diseases, such as CF, which is associated with the production of viscous and adherent mucus in the airway.^16,17^ Two major mechanisms may stop particles from readily diffusing through this mucus gel, namely, “size filtering” through the mucus meshes and “interaction filtering”.^15,18^ Insoluble particles may be trapped in the mucus gel layer or establish hydrophobic, electrostatic, and/or hydrogen bonding interactions with the negatively charged mucin chains.^18,19^ This issue needs to be properly considered when designing novel inhaled siRNA nanomedicines.

To understand in depth the potential of hNPs for siRNA delivery to the lungs, here, we investigated the behavior and the interactions of hNPs with mucus. In a translational perspective, we encapsulated inside hNPs an siRNA pool against the nuclear factor-κB (NF-κB), coordinating the *in vivo* expression of several genes involved in acute and chronic inflammation,^20,21^ also in CF.^22^ Since PEGylated nanoparticles are at the cutting edge to overcome the mucus barrier,^23^ we examined the potential of PEGylated hNPs, which have been investigated so far for reasons other than siRNA lung delivery.^24−27^ Thus, siRNA loaded hNPs were prepared with a PLGA core and coated by either non-PEGylated or PEGylated lipid shells, employing dipalmitoyl phosphatidylcholine (DPPC) or *N*-(carbonyl-methoxypolyethyleneglycol-2000)-1,2-distearoyl-*sn*-glycero-3-phosphoethanolamine (DSPE-PEG). Polyethylenimine (PEI) was introduced as an additional component in hNPs due to its proven ability to interact with siRNA forming a complex able to improve its loading inside hNPs.^12^ hNPs loaded with a fluorescent siRNA were tested for release and transport properties in artificial mucus and sputum from CF patients. Crucial information on hNP properties governing their interaction with CF mucus were acquired by small-angle X-ray scattering (SAXS) studies. Finally, the *in vitro* proof of concept of the ability of siRNA-loaded hNPs to penetrate inside mucus-lined lung epithelia is demonstrated by using a cell culture model based on mucus-covered confluent Calu-3 monolayers.

## Experimental Section

### Materials

The Resomer RG 502H (uncapped PLGA 50:50, inherent viscosity 0.16 0.24 dL/g) was purchased from Evonik Industries AG (Germany). Rhodamine-labeled PLGA was purchased from PolySciTech (USA). 1,2-Dipalmitoyl-*sn*-glycero-3-phosphocholine (DPPC) and 1, 2-distearoyl-*sn*-glycero-3-phosphoethanolamine-Poly(ethylene glycol) (DSPE-PEG) were a kind gift of Lipoid GmbH (Switzerland). Dharmacon ON-TARGETplus siRNA pools against p50 (SMARTpool Nfkb1 siRNA) and p65 (SMARTpool RelA siRNA) NF-kB subunits and ON-TARGETplus non-targeting pool (non-coding siRNA or siNC) were purchased from Carlo Erba Reagents s.r.l. (Italy). Alexa Fluor 647–labeled siRNA (siFluo) was obtained by labeling amine-modified siRNA (Integrated DNA Technologies, Leuven, Belgium) with Alexa Fluor 647 (Life Technologies, Darmstadt, Germany) according to the manufacturer’s protocol and purified by ethanol precipitation and spin column binding as described previously.^28^ Egg yolk emulsion, deoxyribonucleic acid sodium salt from calf thymus (DNA), diethylenetriaminepentaacetic acid (DTPA), phosphate buffer salts, branched polyethylenimine (PEI; 25 kDa), potassium chloride, RPMI 1640 amino acid solution, sodium chloride, Eagle’s Minimum Essential Medium (EMEM), fetal bovine serum and sterile 1× PBS, lipopolysaccharides from *Escherichia coli* O111:B4 (LPS), 3-(4,5-dimethylthiazol-2-yl)-2,5-diphenyltetrazolium bromide (MTT), and RIPA buffer were purchased from Sigma-Aldrich (Missouri, USA). Methylene chloride and ethanol 96% (v/v) were supplied by Carlo Erba (Italy). Penicillin/streptomycin and trypsin 0.25% were purchased from Thermo Fisher Scientific (Waltham, Massachusetts USA). Cell culture flasks (75 cm^2^) and multiwell plates were purchased from Greiner Bio-One (Kremsmünster, Austria). Transwell permeable supports were from Corning (NY, USA). PneumaCult ALI medium, hydrocortisone, and heparin were purchased from STEMcell technologies (Vancouver, Canada).

### Production of siRNA-Loaded hNPs

PLGA-based hNPs loaded with an siRNA pool against NF-kB were prepared by an emulsion/solvent diffusion technique, as described previously.^12^ Briefly, a water solution containing siRNA (100 μL; 0.1 nmol/mL) was emulsified by vortex mixing (2400 min^–1^, Heidolph, Germany) in methylene chloride containing PLGA (1 mL; 1% w/v) and the selected lipid (DPPC/PLGA or DSPE-PEG/PLGA 1:20 w/w). Where indicated, PEI was added to the internal water phase (0.016 mg per 100 mg of PLGA). Just after mixing, the w/o emulsion was added to ethanol 96% (12.5 mL) under moderate magnetic stirring, leading to the immediate precipitation of hNPs. The formulation was diluted with 12.5 mL of Milli-Q water and maintained under stirring for 10 min. Afterward, residue organic solvent was removed by rotary evaporation under vacuum at 30 °C (Heidolph VV 2000, Germany) down to a final volume of 5 mL. hNPs were isolated by centrifugation at 7000 rcf for 20 min at 4 °C (Hettich Zentrifugen, Germany) and dispersed in Milli-Q water. D_2_O was used for H1-NMR.

Fluorescently labeled hNPs were prepared using rhodamine-labeled PLGA (PLGA-Rhod) (resulting formulations: NFkB_DPPC_Rhod_, PEI/siNFkB_DPPC_Rhod_, PEI/siNFkB_DSPE-PEG_Rhod_, PEI/siNFkB_DSPE-PEG_Rhod_,) in the organic phase at 10% w/w with respect to the total PLGA amount. Alternatively, fluorescent hNPs were obtained encapsulating a fluorescent siRNA labeled with AlexaFluor647 (siFluo) (resulting formulations: siFluo_DPPC, PEI/siFluo_DPPC, siFluo_DSPE-PEG, PEI/siFluo_DSPE-PEG).

### Characterization of siRNA-Loaded hNPs

The hydrodynamic diameter (*D_H_*), polydispersity index (PDI), and zeta potential (ζ potential) of hNPs in water were determined by dynamic light scattering (DLS) and electrophoretic light scattering (ELS) (Zetasizer Nano ZS, Malvern Instruments Ltd., UK). Results are expressed as the mean value ± standard deviation (SD) of triplicate measurements on different batches.

The morphology of the hNPs was evaluated by transmission electron microscopy (TEM) with an FEI Tecnai G2 200 kV s-Twin microscope equipped with a 4K camera (ThermoFisher Scientific). Sample analysis was performed upon air drying of 10 μL hNPs dispersions in water (3 mg/mL) mounted on 200 mesh copper grids coated with carbon film (Ted Pella Inc., Nanovision, Italy).

The fixed aqueous layer thickness (FALT) was evaluated by monitoring the influence of ionic strength on particle surface charge.^29^ To this purpose, different amounts of a stock NaCl solution were added to a 0.5 mg/mL hNP aqueous dispersion, and the ζ-potential of the samples was recorded.

^1^H NMR analysis was performed at 500 MHz with a Bruker FT-NMR DRX500 spectrometer using a 5 mm z-PFG (pulsed field gradient) broadband reverse probe in D_2_O at 298 K. Chemical shifts were reported as δ(ppm) relative to residual HDO fixed at 4.70. Diffusion experiments were carried out with the Bruker sequence LEDBPGP2s (DOSY in the following) using stimulated echo and LED with bipolar gradient pulses for diffusion and two spoil gradients. The acquisition was done ranging from 0.674 to 32.030 G/cm with 32 increments using a gradient pulse duration (δ) from 3.2 to 4.4 ms and a diffusion time (Δ) from 100 to 250 ms. The eddy current delay and the gradient recovery delay were 5.0 and 0.2 ms, respectively. hNP dispersions in D_2_O (10 mg/mL) were analyzed. For comparison, dispersions in D_2_O of DSPE-PEG (0.5 mg/mL) and nanoparticles based on PLGA-PEG2000 (10 mg/mL) were analyzed. After diffusion experiments, a solution (10 μL) of 0.538 M dimethylsulfoxide in D_2_O was added to the samples (0.53 mL) as a quantitative reference.

### siRNA Loading inside hNPs

siRNA actual loading was evaluated indirectly by quantitation of non-encapsulated siRNA. Briefly, just after production, hNPs were collected by centrifugation (7000 rcf for 20 min at 4 °C) and the supernatant was analyzed for siRNA content using Quant-IT RiboGreen reagent (Thermo Fisher Scientific, Massachusetts, USA) according to the manufacturer’s instructions. Quantitative analysis was performed by spectrofluorimetry at λ_ex_/λ_em_ 480 nm/520 nm (ProMega GloMax Plate reader, USA). Results are reported as actual loading (nmol of encapsulated siRNA per mg of hNPs) and encapsulation efficiency (actual loading/theoretical loading × 100) ± SD of values collected from three different batches.

### Interactions of hNPs with Mucin and Artificial Mucus

hNPs/mucin interactions were determined in mucin from porcine stomach, Type II (Sigma-Aldrich, Merk KGaA, Darmstadt, Germany), and artificial CF mucus (AM). AM was prepared as previously reported.^30^ Briefly, 25 μL of sterile egg yolk emulsion, 25 mg of mucin from porcine stomach, Type II, 20 mg of DNA, 30 μL of aqueous DTPA (1 mg/mL), 25 mg NaCl, 11 mg KCl, and 100 μL of RPMI 1640 were added to 5 mL of water, and the dispersion was stirred until a homogenous mixture was obtained.

The light scattering of hNP aqueous dispersions with or without mucin was assessed by spectrophotometry as previously described.^29^ Briefly, a saturated solution of Type II mucin was prepared dispersing an excess of mucin in water (0.08% w/v), under stirring overnight, followed by centrifugation at 6000 rcf, 4 °C for 20 min and collection of the mucin-containing supernatant. Then, 10 μL of hNPs dispersion in water were diluted to 1 mg/mL in the mucin solution. The light scattering of the mucin/hNPs mixtures was measured by spectrophotometric analysis at 650 nm at time 0 and after incubation for 30 and 60 min at room temperature. Reference absorbance of mucin and 1 mg/mL hNP dispersions in water were also evaluated. Experiments were run in triplicate, and results are expressed as absorbance at 650 nm ± SD over time.

hNP/mucin interactions were further probed by DLS, comparing the size of hNP dispersion in water, mucin, and AM.^31^ A 10-fold dilution of AM in water was employed for DLS analysis. Data were reported as mean diameter ± SD calculated on three different batches.

### Cellular Mucus (CM) Sampling and Treatment

CM samples were obtained by collection of the secretion produced by human primary bronchial epithelial cells (HBEC) derived from CF patients homozygous for F508del mutation and from non-CF subjects (hereinafter referred to as wild type, *WT*) obtained from “Servizio Colture Primarie” of the Italian Cystic Fibrosis Research Foundation (U.O.C. Genetica Medica, IRCSS Istituto Giannina Gaslini, Genoa, Italy). Cells were cultured on Transwell permeable supports (6 well inserts, 0.4 μm pore size) and allowed to differentiate on an air–liquid interface (ALI) to achieve a fully differentiated muco-ciliary airway epithelium as previously reported.^32^ After 4 weeks of ALI-cultures, mucus secretion was harvested once a week by washing the apical surface with D-PBS (300 μL per 24 mm diameter well insert) followed by 30 min incubation at 37 °C before removal. After low-speed centrifugation of cells and larger debris (300 × *g* for 5 min), mucus from 6-well inserts was pooled and stored at −80 °C until SAXS analysis.

### Small-Angle X-ray Scattering (SAXS) Studies

Synchrotron SAXS measurements were performed at the ID02 high-brilliance beamline^33^ at the ESRF (Grenoble, France), with a beam cross section of 200–400 μm and wavelength λ = 0.1 nm, using two different sample detection distances: 1 and 6 m. The range of investigated momentum transfer, *q* = (4π/λ)sin(θ), was 0.0007 nm^–1^ < *q* < 6 nm^–1^, where 2θ is the scattering angle. All measurements were performed at *T* = 25 °C. Samples were put in plastic capillaries (KI-BEAM, ENKI srl) with 2 mm internal diameter and 0.05 mm wall thickness and closed with polyethylene caps. Capillaries were then mounted horizontally onto the sample holder. Sample concentration was 10 mg/mL. The exposure time of each measurement was 0.1 s, and spectra were checked for radiation damage. The measured SAXS profiles report the scattered radiation intensity as a function of the momentum transfer, *q*, where 1 and 6 m sample-to-detector distance spectra were merged. Solvent subtraction was obtained by measuring water-filled capillaries and empty capillaries and subtracting their intensities from the sample’s ones. Data were analyzed in the Guinier–Porod approximation and were fitted by modeling the hNPs as spherical multishells by using the routines developed in the SaSView program (SasView - Small Angle Scattering Analysis, 2019. Available at https://www.sasview.org/). hNP structure and stability was assessed in water and upon contact with either AM or CM.

### CF Sputum Sampling and Characterization

The sputum was recovered from CF patients of the Children’s Hospital Bambino Gesù of Rome (Italy), upon approval of the Ethics Committee (1700/2018). Subjects or their parents gave and signed a written informed consent. A heterogeneous and representative set of CF patients was selected for the study. Data regarding age, sex, CFTR mutation (genotype), ongoing inhaled and/or antimicrobial therapy, pulmonary function (i.e., forced expiratory volume in the first second and forced vital capacity), and lung bacterial colonization were collected from medical records. Samples from sputum induction in non-expectorating children and spontaneously expectorated sputum mixed by a magnetic stirrer were exposed to an ultraviolet light-emitting diode (UV-LED). The sputum irradiation at 265 nm for 6 h at 22 °C in a safety cabinet effectively inactivated pathogenic microorganisms (data not shown). After treatment, all samples underwent freezing, which was previously demonstrated to affect neither the viscoelastic properties of native mucus nor drug coefficient of diffusion across mucus models.^34^

DNA quantitation in CF sputa was performed by a Quant-IT PicoGreen dsDNA assay kit according to the manufacturer’s instructions (Thermo Fisher Scientific, Massachusetts, USA). The analysis was performed by spectrofluorimetry at λ_ex_/λ_em_ 480 nm/520 nm (ProMega GloMax Plate reader, USA). A calibration curve was obtained with standard solutions of DNA from calf thymus (Sigma-Aldrich, USA). The linearity of the response was verified over the concentration range 0.001–10 μg/mL (*r*^2^ ≥ 0.99). The results are reported as μg/mL of DNA ± SD of three different measurements.

Quantitation of human Mucin 5 subtype B (MUC5B), Mucin 5 subtype AC (MUC5AC), and Mucin 2 (MUC2) was performed by specific enzyme-linked immunosorbent assay (ELISA) kits according to the manufacturer’s procedure (Elabscience Biotechnology Inc., USA). The optical density (OD) was measured spectrophotometrically at a wavelength of 450 nm (ProMega GloMax Plate reader, USA). The individual mucin concentration in each sputum sample was calculated by comparing the OD of the sample with the corresponding calibration curve (reference standards 0.156–10 ng/mL; *r*^2^ > 0.99). The results are reported as ng/mL of mucin ± SD of three different measurements.

### *In Vitro* Transport of hNPs through Artificial Mucus and CF Sputum

A previously developed model based on Transwell multiwell plates^29^ was used for diffusion experiments. Briefly, 75 μL of artificial mucus or CF sputum were transferred into each Transwell insert (6.5 mm; pore size: 8 μm). Afterward, 25 μL of a hNP dispersion in water (20 mg/150 μL) were placed on top of the medium and the insert was transferred in a 24-well plate containing 300 μL of simulated interstitial lung fluid (SILF) per well. SILF (NaCl 0.1 M, KCl 4 mM, NaHPO_4_ 1 mM, Na_2_SO_4_ 0.5 mM, CaCl_2_ 2.5 mM, MgCl_2_·6H_2_O 1.5 mM, NaHCO_3_ 31 mM, sodium acetate 7 mM, sodium citrate monohydrate 0.36 mM) was prepared as previously reported.^35^

At scheduled time intervals, the acceptor medium was sampled and centrifuged at 9000 rcf for 20 min at 4 °C to isolate hNPs. The pellet was suspended in 50 μL of water, diluted with 450 μL of 0.5 M NaOH, and stirred for 1 h to degrade the PLGA matrix. The amount of hNPs in the resulting solution was quantified by spectrofluorimetric analysis of PLGA-Rhod at λ_ex_ 520 nm/λ_em_ 580–640 nm (GloMax Explorer, Promega, Italy). Calibration curves were derived by analyzing serial dilutions of standard solutions prepared from an hNP stock degraded in 0.5 M NaOH. The linearity of the response was verified over the concentration range 2–200 μg/mL (*r*^2^ ≥ 0.999). Experiments were run in triplicate, and the results were expressed as percentage (%) of total hNPs permeated over time ± SD.

### hNP Uptake in Mucus-Covered Calu-3 Cell Monolayers

Calu-3 cells were seeded in a clear polyester cell culture insert (growth area 1.12 cm^2^, pore size 0.4 μm) on Transwell permeable supports. Per sample, 500,000 cells were seeded in 500 μL of medium in the apical chamber, while 1.5 mL of the medium was added to the basolateral chamber. After 72 h, the medium in the upper and basolateral chamber was removed and replaced by fresh Pneumacult ALI medium (Stemcell Technologies GmbH, Germany) to air-lift the culture. Once TEER values ≥300 Ω·cm^2^ were reached and a stable polarized epithelial layer was formed, cell layers were used for cell uptake experiments and mucus transport studies.

For cell uptake experiments, Calu-3 monolayers were incubated with 500 μL of PEI/siFluo_DPPC or PEI/siFluo_DSPE-PEG resuspended in medium corresponding to a 20 nM siRNA final concentration (10 pmol siFluo per well). After 24 h, monolayers were washed, cells were detached from the inserts and the dispersion was analyzed by flow cytometry on an Attune NxT flow cytometer (Thermo Fisher Scientific) with 638 nm excitation and a 670/14 nm emission filter. All cells were gated according to morphology based on forward/sideward scattering, and 10,000 events were evaluated per sample to determine the median fluorescence intensity (MFI) and siFLuo uptake per cell. Controls consisted of cells transfected with free siFluo and lipofectamine/siFluo complexes as negative and positive controls, respectively.

For transport experiments across the mucus layer, differentiated mucus-covered Calu-3 cells were exposed for 24 h to PEI/siFluo_DPPC or PEI/siFluo_DSPE-PEG amounts corresponding to 10 pmol siFluo per well. Monolayers were then washed with PBS, and the mucus layer was stained by incubation with AlexaFluor488-labeled wheat germ agglutinin (10 μg/mL) (Invitrogen, Thermo Fisher Scientific, USA) for 10 min at 37 °C. After washing with PBS, the membranes of the Transwell inserts were cut, mounted on microscope slides, covered with coverslips, and immediately analyzed by confocal laser scanning microscopy (CLSM) (SP8 inverted scanning confocal microscope, Leica Camera, Wetzlar, Germany). Z-stack pictures were taken, and optical sections were processed to create a 3D view, which allows observing the diffusion of the hNPs through the mucus and their cellular internalization.

### *In Vitro* NFκB Gene Silencing in LPS-Stimulated 16HBE14o- Cells

Epithelial 16HBE14o- cells were grown in EMEM supplemented with 1% l-glutamine, 1% penicillin/streptomycin, and 10% fetal calf serum at 37 °C in a 5% CO_2_ humidified atmosphere. Cells were passaged every 3–4 days once reached confluency. Afterward, 2.5 × 10^4^ cells were seeded in 6-well plates with 2.5 mL of medium and incubated overnight. Before transfection, cells were stimulated with LPS (25 μg/mL) to induce NFκB gene expression. After 4 h, the medium was removed, and the cells were transfected with hNPs containing 20 nM siNFκB or non-coding siNC for 72 h at 37 °C in a 5% CO_2_ humidified atmosphere. siRNA/lipofectamine complexes were used as positive controls. After transfection, cells were lysed using RIPA Buffer with 1× cocktail of protease/phosphatase inhibitors (Roche, Basel, Switzerland). The samples were analyzed for the expression of NFκB p65 subunit (NFκB p65 mouse monoclonal antibody, 1:500, sc-1008, Santa Cruz Biotechnology) by Western blot analysis, following a published protocol.^36^ Actin (actin goat polyclonal IgG, 1:500, Santa Cruz Biotechnology) was used as housekeeping protein. Mouse IgG binding protein conjugated to horseradish peroxidase (mIgG BP-HRP) and donkey anti-goat IgG-HRP were used as secondary antibodies (1:5000, Santa Cruz Biotechnology). Membranes were treated with SuperSignal West Pico PLUS chemiluminescent substrate (Thermo Fisher Scientific) immediately before analysis at the ChemiDoc MP imaging system (Bio Rad). Cell studies were performed in triplicate, and results are expressed as mean value ± SD, *n* = 3. One-way ANOVA, **p < 0.01, ***p < 0.005.

## Results and Discussion

### Overall Properties of siRNA-Loaded hNPs

**Figure 1:**
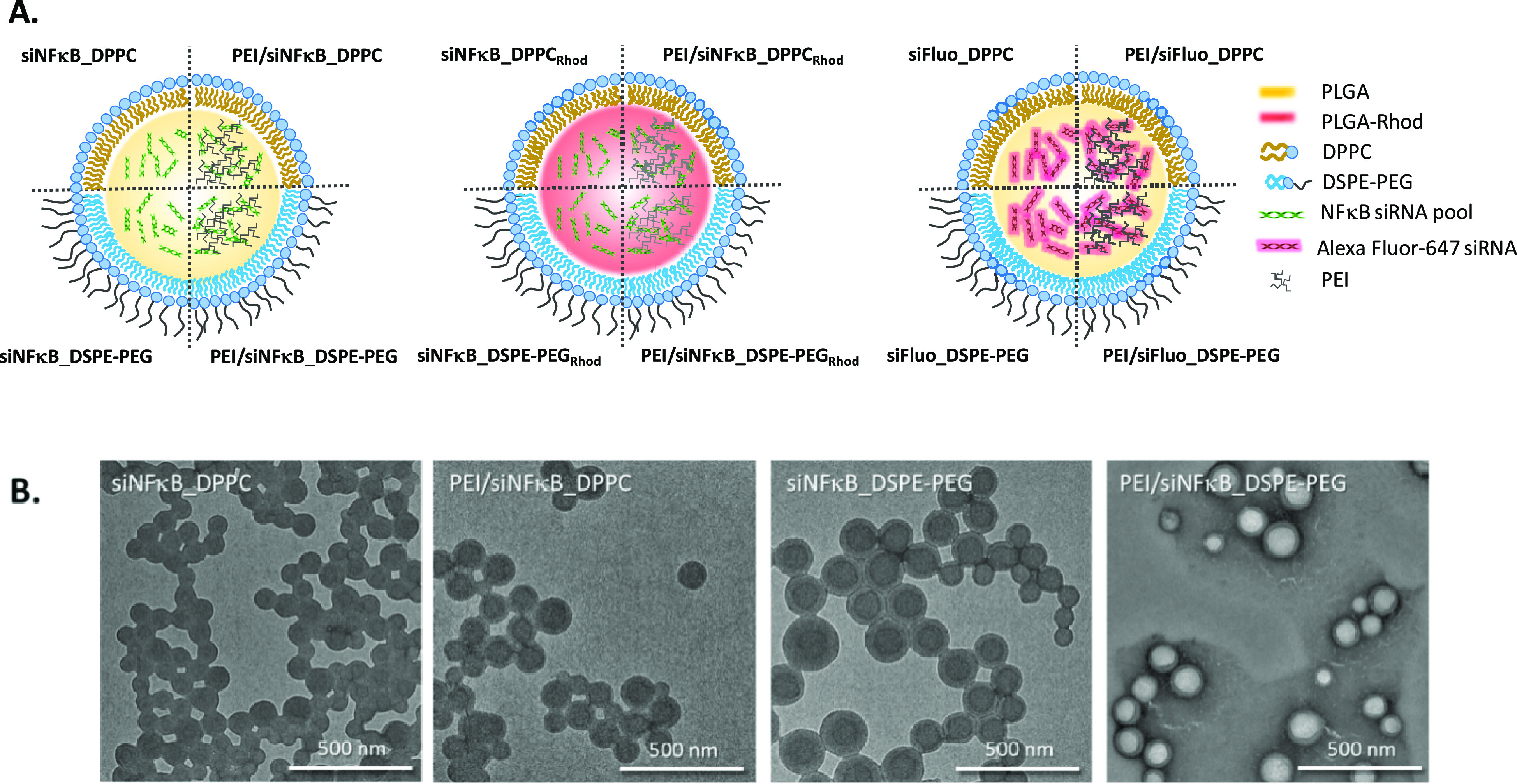
Optimized siRNA-loaded hNPs. (A) Sketched representation of the hNP structure and composition. (B) Transmission electron microscopy (TEM) image of the hNPs (the field is representative of the formulation).

hNPs containing an siRNA pool against NFkB (siNFkB) were successfully produced by an emulsion/solvent diffusion technique employing two different lipids as surface modifiers, that is DPPC and DSPE-PEG, in the presence (i.e., PEI/siNFkB_DPPC and PEI/siNFkB_DSPE-PEG hNPs, respectively) or absence of PEI (i.e., siNFkB_DPPC and siNFkB_DSPE-PEG hNPs, respectively) as the fourth component of the PLGA nanoparticulate system. To quantify the amount of hNPs transported through mucus, corresponding fluorescently labeled hNPs were prepared using PLGA-Rhod (i.e., siNFkB_DPPC_Rhod_, siNFkB_DSPE-PEG_Rhod_, PEI/siNFkB_DPPC_Rhod_, PEI/siNFkB_DSPE-PEG_Rhod_). For transport experiments, hNPs containing a fluorescent Alexa Fluor-647 siRNA (siFluo) were employed. A sketched representation of the adopted concepts is shown in Figure 1 A.

As can be seen in Table 1, the formulation conditions do not significantly affect hNPs properties. Independently of the lipid shell type and the presence of PEI inside the formulation, siNFkB-loaded hNPs show negative ζ potential, D_H_ lower than 180 nm and low PDI values (ranging from 0.06 to 0.2). TEM images indicate the formation of regularly shaped hNPs displaying a halo, likely compatible with a core shell structure in which the PLGA core, containing siNFkB or PEI/siNFkB, is covered by the lipid shell (DPPC or DSPE-PEG) (Figure 1B).

**Table 1:** Size, ζ Potential and Encapsulation Efficiency of the Optimized siNFκB-Loaded hNPs^a^

Formulation	*D_H_* (nm ± SD)	PDI (mean ± SD)	SAXS *R_G_* (nm)	ζ potential (mV ± SD)	YP (%)	EE (%)	AL (nmol/100 mg hNPs)
siNFkB_DPPC	177.6 ± 9.2	0.193 ± 0.039	68.0 ± 0.6	–28.7 ± 1.6	86 ± 6	60 ± 1	0.599 ± 0.004
PEI/siNFkB_DPPC	161.3 ± 3.8	0.170 ± 0.042	67.0 ± 0.7	–23.3 ± 0.7	71 ± 4	91 ± 4	0.914 ± 0.038
siNFkB_DSPE-PEG	159.1 ± 5.1	0.062 ± 0.017	81.9 ± 0.2	–28.6 ± 1.7	80 ± 1	80 ± 2	0.796 ± 0.022
PEI/siNFkB_DSPE-PEG	165.5 ± 14.1	0.105 ± 0.069	72.1 ± 0.4	–28.9 ± 6.9	64 ± 3	89 ± 6	0.894 ± 0.064

a *D_H_* = hydrodynamic diameter. PDI = polydispersity index. YP = yield of production. EE = encapsulation efficiency. AL = actual loading. *R_G_* = gyration radius from Guinier’s analysis.

SAXS investigation of hNPs confirms that finite size particles, in the size range of 100–150 nm, are suspended in the dispersant. The measured Porod limit showing a *q*^–4^ power law identifies a sharp interface and a smooth surface, suggesting that the inner PLGA core, structured as a network with a fractal geometry,^37^ is coated at the surface by the smooth phospholipid layer. Guinier and Fournet’s analysis^38^ of the lowest *q*-vector points gives a measure of the gyration radii (*R_G_*), which are in line with DLS measurements (Table 1). As previously observed with DPPC-engineered hNPs,^12^ the best fits of SAXS spectra of siRNA-loaded hNPs were obtained by applying the form factor of polydispersed spheres with a spherical radius *R_S_* (Figure S1A). We found Rs values of 70 ± 21 and 61 ± 18 nm (mean ± SD) for siNFkB_DSPE-PEG and PEI/siNFkB_DSPE-PEG, respectively (polydispersity ratio of 0.3). The scattering profiles were reproduced as well by a spherical core-multishell form factor with polydispersed thicknesses, satisfying the electron density profiles expected for the formulation of PLGA, and a phospholipid shell of thickness 2.4 ± 0.2 nm (Figure S1B). Nonetheless, the PEG external shell showed a very feeble electron density difference with respect to the solvent and is hardly visible. Of course, as the number of parameters increases a lot with respect to those of a simple spherical form factor, we can only state the agreement of the more complex fitting to avoid overfitting hazard. As expected from previous studies,^12^ PEI is able to complex siNFκB (Figure S2), thus increasing the amount of siRNA entrapped inside the hNPs (Table 1). A 30% increase of the EE was observed for PEI/siNFkB_DPPC hNPs as compared to siNFkB_DPPC, while a slighter change in EE was achieved when adding PEI in hNPs prepared from DSPE-PEG/PLGA mixtures (from 80% of siNFkB_DSPE-PEG to 89% of PEI/siNFkB_DSPE-PEG).

The presence of a PEG shell on the surface of DSPE-PEG modified hNPs was suggested by FALT measurements performed by monitoring the influence of ionic strength of the dispersing medium on particle surface charge (Figure S3). According to the Gouy–Chapmann theory, the ζ-potential decreases with the increase of the ionic strength of the medium. In agreement with SAXS, data fitting by linear regression indicates that a shell around 2 nm (1.74–1.87 nm) is formed on the surface of the hNPs when DSPE-PEG was used as a lipid component, independently on the presence of PEI (Figure S3A). Contrariwise, the ζ-potential of control non-pegylated DPPC-based hNPs does not increase when increasing the ionic strength of the medium (Figure S3B).

To gain information on the PEG aggregation state, parallel ^1^H NMR experiments were performed on D_2_O dispersions of (a) PEI/siNFkB_DSPE-PEG, (b) PEI/siNFkB_DPPC, (c) PEI/siNFkB-loaded nanoparticles based on PLGA-PEG2000 (PEI/siNFkB_PLGA-PEG), and (d) DSPE-PEG lipid, for the sake of comparison. The low mobility of large aggregates prevents the detection of high-resolution NMR signals, while small aggregates and molecules produce a spectrum. A high-resolution PEG peak at 3.62 ppm is visible in all samples, except for PEI/siNFkB_DPPC, and internal reference allowed to estimate their molarity (Figure S4). PEI/siNFkB_DSPE-PEG hNPs show a sharp peak, while PEI/siNFkB_PLGA-PEG nanoparticles, with a *D_H_* of 110 ± 10 nm, display the overlapping of a sharp and a broad component. The same sharp component is visible in DSPE-PEG dispersion with 7.7 times higher intensity (Figure S4). DOSY diffusion experiments indicate that the sharp PEG signal does not belong to the same aggregate size in the three samples (Figure S5). The diffusion coefficients were derived, giving *D* values of (2.78 ± 0.09)·10^–11^ m^2^/s, (1.12 ± 0.04)·10^–10^ m^2^/s, and (2.625 ± 0.005)·10^–11^ m^2^/s for PEI/siNFkB_DSPE-PEG hNPs, PEI/siNFkB_PLGA-PEG, and DSPE-PEG dispersions, respectively. Control diffusion experiments were performed on mePEG750 and PEG2000 in D_2_O and on residual HDO signal in D_2_O for gradient calibrations (data not shown). Appling the Stokes–Einstein relation, we estimate that the residual PEG signal in PEI/siNFkB_DSPE-PEG hNPs and DSPE-PEG control dispersions belongs to aggregates with a 9 nm radius corresponding to the size of DSPE-PEG lipid micelles in water.^39^ The molar ratio between the PEG signals in the dispersions show that PEI/siNFkB_DSPE-PEG hNPs contain a 13% of DSPE-PEG solved in micellar form with a radius of about 9 nm, in equilibrium with a much larger proportion (87%) on the hNPs.

### Behavior of siRNA-Loaded hNPs in Mucin Dispersions and Artificial CF Mucus

**Figure 2:**
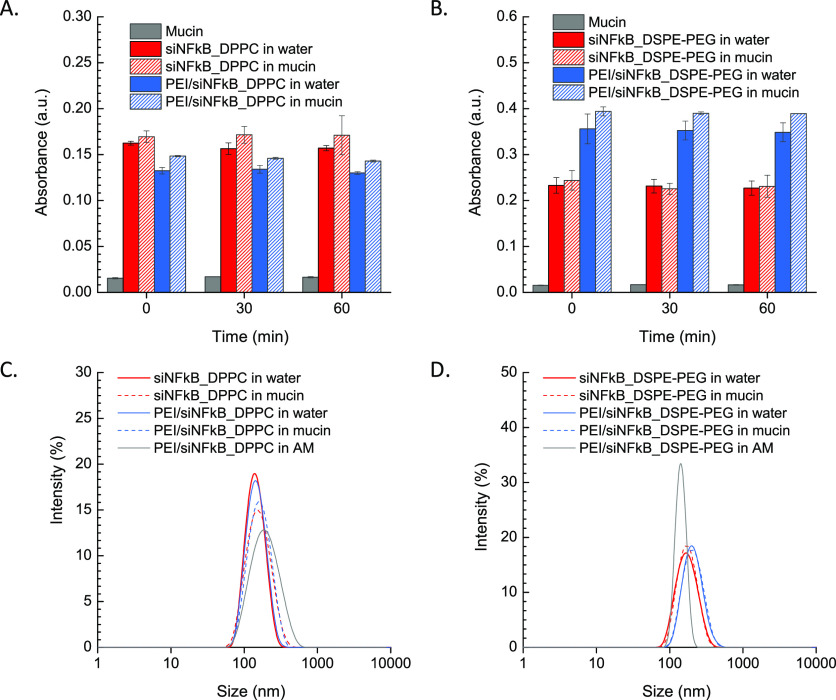
*In vitro* assessment of siNFkB-loaded hNPs interactions with mucin and artificial CF mucus (AM). (A,B) Scattering at 650 nm of hNPs (1 mg/mL) in the presence of mucin over time. Scattering at 650 nm of hNP dispersions in water are reported as controls. (C, D) Size distribution by intensity of hNPs in the presence of mucin or AM evaluated by DLS. The size distribution profiles of hNP dispersions in water are reported as controls. Representative results achieved for PEI-containing hNPs are reported in AM.

A critical point in defining particle ability to diffuse across the mucus layer is the evaluation of the interactions between particles and mucus components. The absence of such interactions is commonly assumed as the *conditio sine qua non* for an efficient mucus-penetrating nanoparticulate system.^5^ To this aim, the interactions between hNPs and mucin, one of the main components of airway mucus, were first roughly determined by measuring the scattering at 650 nm of hNP aqueous dispersions with and without mucin. As shown in Figure 2A,B, all formulations displayed the same absorbance at 650 nm in water and in mucin, suggesting no mucin absorption on the hNP surface, independently of the type of lipid employed (i.e., DPPC or DSPE-PEG) or the presence of PEI.

The absence of hNPs/mucin interactions was confirmed when monitoring hNP size in the presence of mucin using DLS (Figure 2C,D). A very slight increase in particle size and a slight broadening in the size distribution was observed only for DPPC-coated hNPs in mucin as compared to the corresponding aqueous dispersions (Figure 2C). This effect was even more evident in AM (*D_H_* = 221.3 ± 1.4 nm and PDI = 0.211 for PEI/siNFkB_DPPC in AM), where a contribution of salts, likely affecting the mucin structure,^40^ and DNA fibers^18,41^ cannot be excluded. Nevertheless, no change was apparent for particles coated with DSPE-PEG, displaying an even lower mean *D_H_* (162.4 ± 3.1 nm) as compared to water (Figure 2D). No significant differences were observed between hNPs with or without PEI in AM (data not shown).

**Figure 3:**
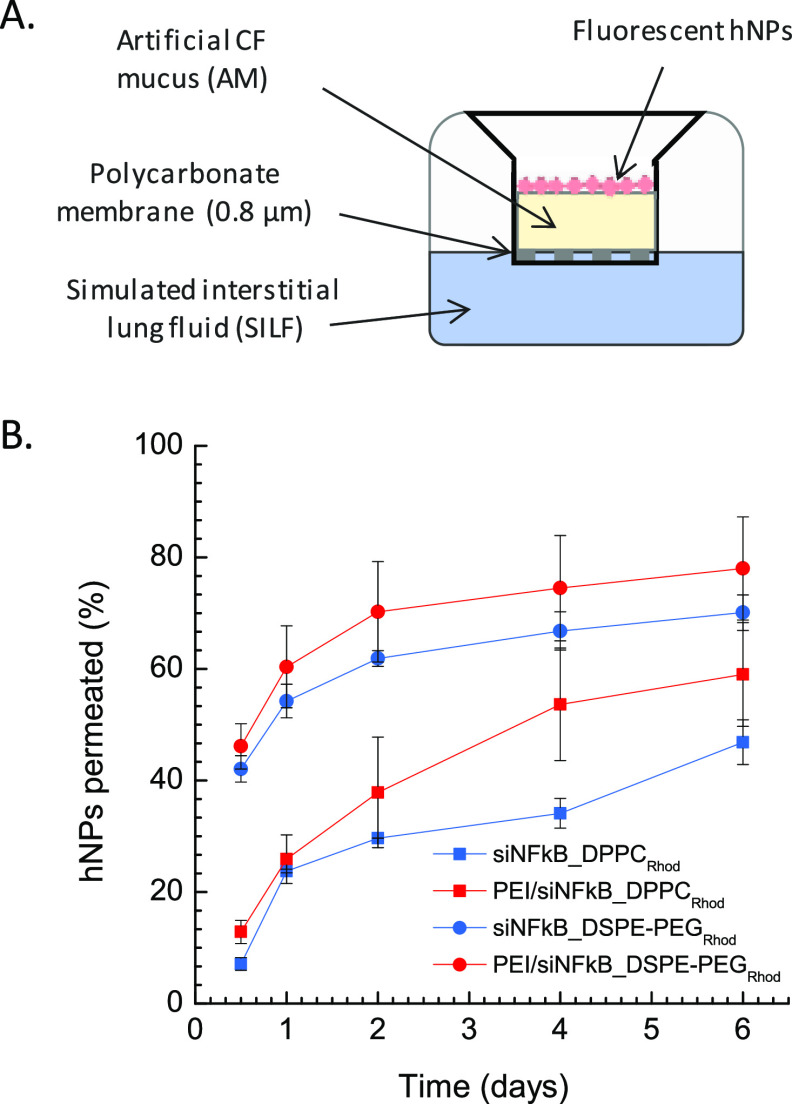
(A) Schematic representation of a Transwell multi-plate assay. (B) Percent amount of siNFkB-loaded fluorescent hNPs permeated through AM in SILF over time. Data are mean values of triplicate experiments ± standard deviation (SD).

To understand the detailed role of AM components on the behavior of hNPs in mucus, the transport of fluorescent siRNA-loaded hNPs through AM was assessed by a Transwell multi-plate assay as previously reported (Figure 3A).^29^ In line with previous literature data,^23^ our results suggest that PEGylated hNPs allow for faster transport through AM as compared to non-PEGylated ones, with about 80% of the total amount of PEI/siNFkB_DSPE-PEG_Rhod_ hNPs permeated after 6 h versus 60% of PEI/siNFkB_DPPC_Rhod_ found in SILF at the same time intervals. As expected from hNPs/AM interaction studies, no significant difference was apparent between hNPs with or without PEI (Figure 3B).

### Behavior of siRNA-Loaded hNPs in Artificial CF Mucus and Mucus Secreted from CF Human Bronchial Epithelial Cells

In order to implement knowledge of hNPs features relevant for pulmonary delivery, the hNP structure and stability in contact with mucus was studied by SAXS analysis in AM, cellular mucus (CM) secreted from CF, and wild type (WT) HBEC grown at ALI.

**Figure 4:**
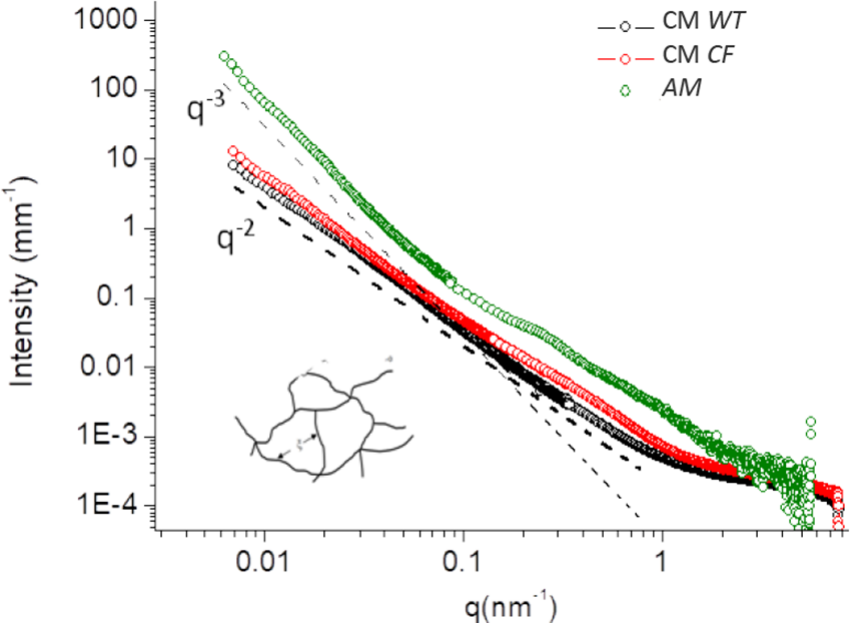
SAXS spectra of cellular mucus (CM) collected from CF and wild type (WT) HBEC grown at ALI in comparison with artificial CF mucus (AM).

The SAXS spectra of CM (both WT and CF) are reported in Figure 4 in comparison with those of AM. The scattering profiles for the three investigated networks are typical of semidiluted gels but reveal important differences. The AM matrix has a significantly steeper slope in the low *q*-vector range, proportional to *q*^–3^, while the cellular models show a *q*^–2.2^ slope, denoting a change from the surface fractal behavior, typical of large scale clustering mesh (AM), to a mass fractal dimensionality, characteristic of semidiluted meshes.

In fact, a −3 power law is at the border between mass- fractals and surface-fractals that, in the case of the AM model, denotes the presence of large aggregates, also highlighted by an increased scattering intensity in the low *q*-vector, corresponding to the large distance scale. The overall difference in the scattering intensity among the different systems is possibly originated by their composition. For a similar mucin content, AM is enriched in DNA and lipids, with respect to WT and CF cellular mucus that are not expected to be contaminated by bacteria.

**Figure 5:**
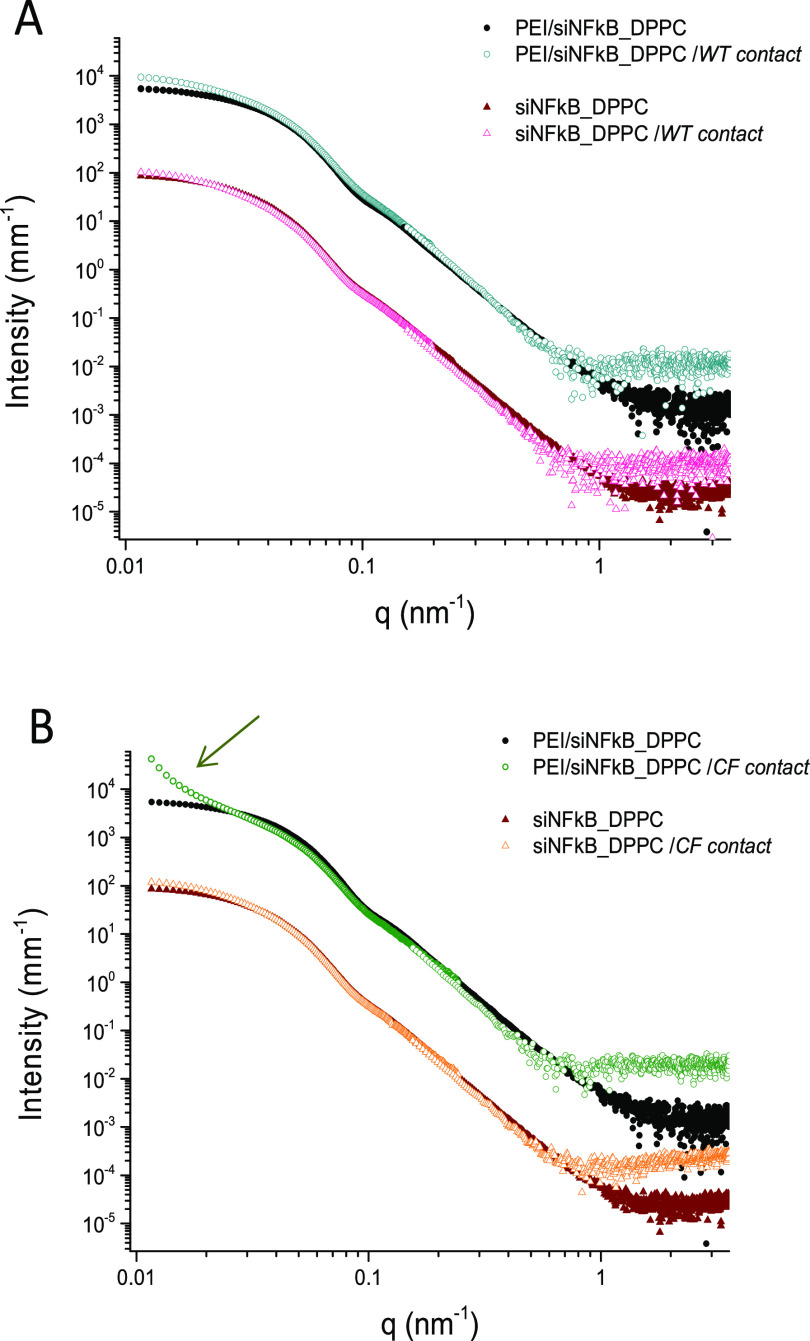
SAXS spectra of siNFkB_DPPC and PEI/siNFkB_DPPC (intensity multiplied by 100) hNPs compared with the profiles obtained after subtracting the cellular mucus spectra from each hNP plus mucus collected from wild type (WT) (A) and CF (B) HBEC grown at ALI. The arrow indicates the presence of large-scale structures induced by PEI/siNFkB_DPPC hNPs in CF cellular mucus. High *q*-vector intensity of the CM contacted systems is interpreted as an extra mass layer coating the hNPs.

As previously observed upon incubation of siRNA-loaded DPPC/PLGA hNPs in AM^12^, SAXS spectra of the mixed systems siNFkB_DPPC plus CM are in general reproducible by a linear combination of the spectra of the bare hNPs and the mucus. In Figure 5, we show that when subtracting the corresponding mucus spectrum from the hNPs plus mucus spectra, a generally good agreement is found with the profile of the original hNPs. This is consistent with a long-time stability of the hNPs in CM. However, when PEI/siNFkB_DPPC hNPs were put in contact with CM from CF cells, we found low-*q* scattering that largely exceeds the scattering of the linear subtraction of the two components (arrow in Figure 5B). This indicates that some particle clustering is induced in the mucus mesh. Importantly, all samples show a higher scattered intensity at high *q*-vectors, where the small features are visible. The profile of the spectra agrees with the presence of extra features sized tenths of nanometers, which, along with the increased intensity at the low *q*-vectors, may account for an extra layer coating the hNPs. This can originate from a partial sequestering of material from the mucus mesh enlarging the size of the particles, as also observed by DLS (see Figure 2C). The effect is more important when interaction occurs with CM from CF cells.

**Figure 6:**
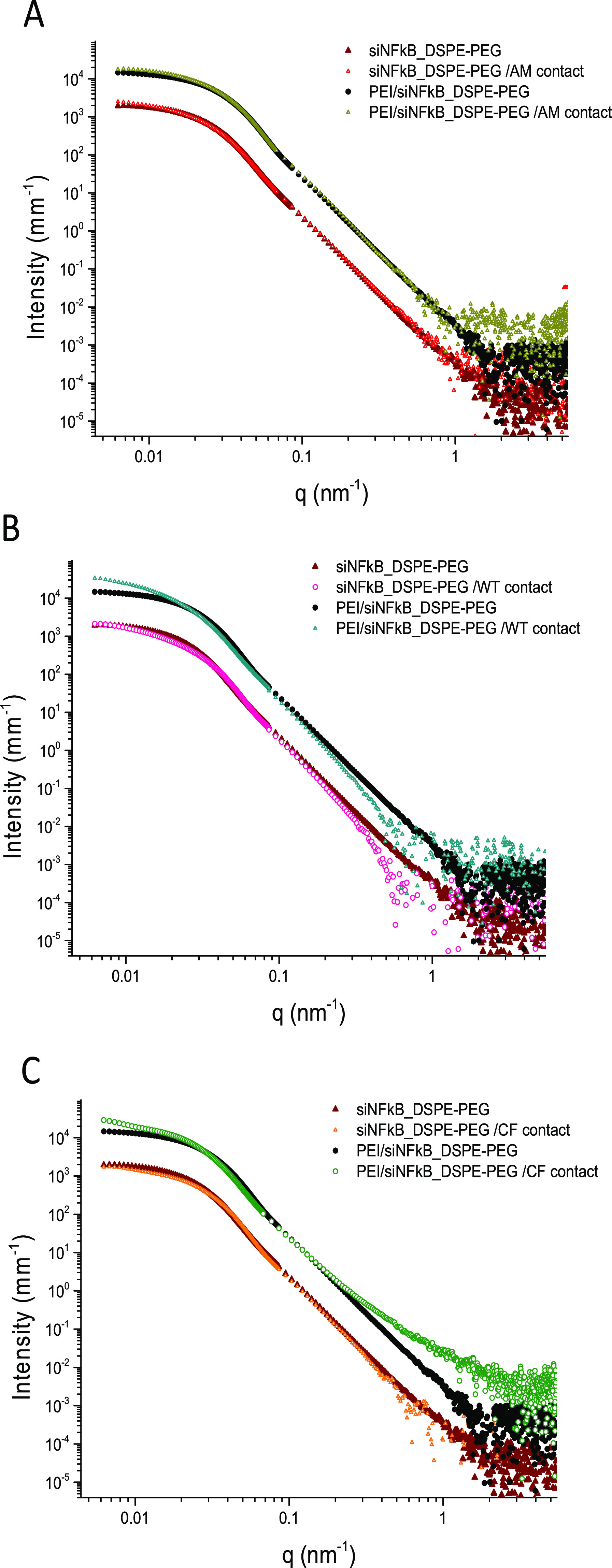
SAXS spectra of siNFkB_DSPE-PEG and PEI/siNFkB_DSPE-PEG (intensity multiplied by 10) hNPs compared with the profiles obtained after subtracting the mucus spectra from each hNPs plus AM (A), cellular wild type mucus (WT) (B), and cellular CF mucus (CF) (C). Mucus effects are more evident on PEI containing hNPs.

The structural outcome regarding the incubation of hNPs modified with DSPE-PEG in AM and CM mucus models are reported in Figure 6. We show the scattering profiles of the bare hNPs and the profiles obtained after subtraction of the correspondent mucus profile from each hNP plus mucus spectrum. No major effect seems to affect the original particles profile, except for an increased intensity at the lowest *q*-vectors of the PEI containing hNPs when in contact with CM. These findings highlight that PEGylated hNPs are not totally inert in CM, when PEI is present in their composition. When PEI/siNFkB_DSPE-PEG hNPs are put in contact with CM from CF cells, the high *q*-vector intensity also increases, likely due to the release of small objects, tenths of nanometers sized, in the dispersion. However, the interaction does not consist any hNP degradation but rather implies that some aggregation process occurs. The preferred interpretation of our finding is given by considering some rearrangement of the mucus network to accommodate the hNPs. In fact, an induced clustering of the mucin matrix could explain the low *q*-vector evidence, suggesting a role of the positively charged PEI polymer as a bridging agent with the negatively charged mucin components. It is not clear why interactions with CM from CF cells are different from the interactions with the healthy control in this respect. Nevertheless, a difference in the CM composition in terms of DNA and mucin contents cannot be excluded,^42^ though previous studies on HBEC derived from non-CF and CF patients revealed no significant differences in the levels of mucins 5-subtype B (MUC5B) and 5-subtype AC (MUC5AC), the major secreted gel-forming mucins.^43^

### Behavior of siRNA-Loaded hNPs in CF Sputum Samples

Results achieved on AM and CM models prompted toward a full understanding of hNP behavior in physiologically relevant settings, that is, human CF sputa. Nowadays, there is a broad consensus that the major consequences of CFTR dysfunction in the airway are dehydration of mucus and reduction in the height of the periciliary layer.^17^ Once infected, CF mucus is infiltrated with neutrophils, contributing to high concentrations of neutrophil-derived DNA and filamentous actin, and microorganisms such as *Pseudomonas aeruginosa*, *Staphylococcus aureus*, and *Aspergillus* species, among others, often in biofilms. All these highly entangled polymeric macromolecules contribute to a firm gel matrix with a pore size reduced from the normal size of approximately 500 to approximately 150 nm.^17,44^

Data regarding age, sex, CFTR mutations, lung colonization/function, and inhaled therapy of enrolled patients are reported in Table 2.

**Table 2:** Overall Features of CF Sputum Samples and Corresponding Donor Patients

					pulmonary function^c^		
sample	sex^a^	age (years)	CFTR mutation	colonization^b^	FEV1	FVC	inhaled therapy^d^
CF 1	M	36	N1303K/M1V	PA+ SA + STM + ECL complex	68%	ND	rhDNase inhalation
CF 2	M	15	F508del/del22,24	PA + SA	74%	90%	rhDNase inhalation
CF 3	M	42	F508del/W1282X	MRSA + AD+ AT + SSP + CoA	30%	57%	rhDNase inhalation plus Meropenem i.v.
CF 4	F	3.5	F508del/F508del	SA	ND	ND	Tobramicyn inhalation
CF 5	F	5.5	F508del/F508del	-	ND	ND	rhDNase inhalation
CF 6	F	17	W1282X/UN	SA+ KD	76%	78%	rhDNase plus Colistin inhalation
CF 7	F	12	G542X/1303 K	PA+ SA+ STM+ BCatar+ ECL complex	101%	102%	rhDNase inhalation
CF 8	M	28	F508del/F508del	PA+ SA+ CPA + CA	111%	115%	rhDNase inhalation
CF 9	F	30	F508del/3272-26A > G	PA+ AF+ CDB+ CTP	21%	34%	rhDNase plus Tobramycin inhalation
CF 10	F	35	F508del/W1282X	PA	79%	98%	rhDNase plus Colistin inhalation
CF 11	F	36	F508del/4016insT	MRSA+ PA+ CA	46%	64%	rhDNase plus Colistin inhalation
CF 12	F	44	F508del/F508del	SA	55%	69%	rhDNase inhalation
CF 13	M	45	F508del/F508del	BC+ EF	88%	89%	rhDNase plus Tobramycin inhalation

a M = male; F = female.

b AD = *Achromobacter denitrificans*; AF = *Aspergillus flavus*; AT = *Aspergillus terreus* ; BC = *Burkholderia cepacia*; BCatar = *Branhamella catarrhalis*; CA = *Candida albicans*; CDB = *Candida dubliniensis*; CoA = *Comamonas acidovorans*; CPA = *Candida parapsilosis*; CTP = *Candida tropicalis*; ECL complex = *Enterobacter cloacaeComplex*; EF = *Enterococcus faecalis*; KD = *Kingella denitrificans*; MRSA = *Meticillin-resistantStaphylococcus aureus*; PA = *Pseudomonas aeruginosa*; SA = *Staphylococcus aureus*; SSP = *Shingobacterium spiritivorum*; STM = *Stenotrophomas maltophilia*.

c FEV1 = forced expiratory volume in the first second; FVC = forced vital capacity; ND = not determined.

d rhDNase = recombinant human deoxyribonuclease; i.v. = intravenous.

**Figure 7:**
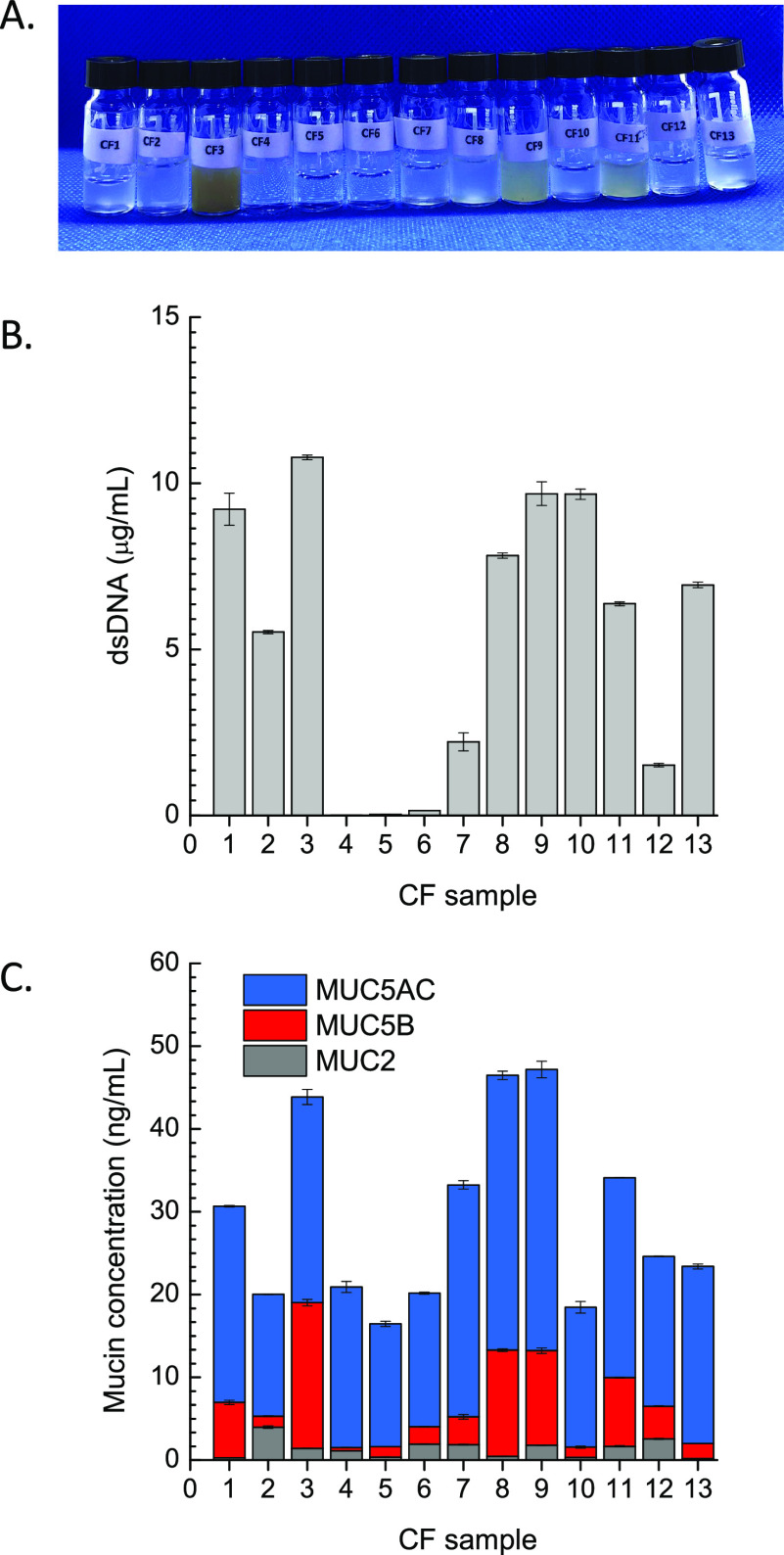
Visual appearance and relevant properties of sputum samples from CF patients: (A) picture taken in a photography light box equipped with a blue backdrops (PULUZ Technology Ltd., China); (B) dsDNA concentration; (C) mucin concentration as a result of MUC2 (gray bars), MUC5B (red bars), and MUC5AC (blue bars) individual concentrations. Data are mean values ± standard deviation calculated on triplicate experiments performed on each CF sputum sample.

Regardless of other patient clinical parameters, the samples here can be roughly grouped according to the different numbers of microbial colonies isolated from the corresponding sample in (i) “high colonization” sputa, corresponding to CF1, CF3, CF7, CF8, CF9, and CF11 (isolated microorganisms ≫ 2); (ii) “low colonization” sputa, corresponding to CF2, CF4, CF5, CF10, CF12, and CF13 (isolated microorganisms ≤ 2). Nevertheless, CF sputa were further characterized for the concentrations of dsDNA and total and individual mucins (Figure 7), which are the major contributors to CF mucus barrier properties.^44^

As stated above, elevated extracellular DNA levels, mainly derived from neutrophils, are commonly found in sputa from CF patients, with an obvious effect on mucus viscosity and elasticity and consequent airway obstruction.^44−46^ Though all the CF individuals contributing to the study samples received inhaled rhDNase, significant DNA concentrations were found in most of the samples, except for CF4, CF5, and CF6 (0.009 ± 0.003, 0.038 ± 0.008, and 0.145 ± 0.005 μg/mL of dsDNA, respectively) (Figure 7A). Even though the highest DNA levels were found in the CF3 sputum (i.e., 10.8 ± 0.070 μg/mL), similar amounts were found also in other samples (9.22 ± 0.485, 9.68 ± 0.357, and 9.67 ± 0.152 for CF1, CF9, and CF10, respectively), suggesting a different contributor to the complexity of the pathological sputa, such as gel-forming mucins.

Literature data suggest that the gel-forming mucins MUC5B and MUC5AC, normally secreted from goblet/mucous cells in healthy airways, are the main determinant of the non-Newtonian properties of airway mucus.^14,44,47^ Nevertheless, the intestinal mucin MUC2 is also reported in small amounts in the CF airways.^42 44,47^ Thus, MUC5AC, MUC5B, and MUC2 amounts in the CF sputum samples were also quantitatively determined by sandwich ELISA relying on biotinylated antibodies specific for human MUC5AC, MUC5B, and MUC2 (blue, red, and gray bars in Figure 7C, respectively). As expected from colonization data, total mucin concentration was significantly higher for “high colonization” samples as compared to “low colonization” ones.

While MUC5AC concentrations within the range 14.7–21.3 ng/mL were recorded for “low colonization” CF samples, MUC5AC levels of ≥24 ng/mL were found in more complex “high colonization” sputa (23.7 ± 0.0915, 24.8 ± 0.915, 28.0 ± 0.519, 33.2 ± 0.519, 34.0 ± 1.01, and 24.2 ± 0.0305 ng/mL of MUC5AC for CF1, CF3, CF7, CF8, CF9, and CF11, respectively). In line with literature data, suggesting an expansion of the MUC5B expression in inflamed CF airways, the contribution of MUC5B to the total mucin concentration was even more evident in “high colonization” sputum samples. Except for sample CF7 (3.36 ± 0.280 ng/mL of MUC5B), samples CF1, CF3, CF8, CF9, and CF11 displayed MUC5B levels within the range 6.70–17.6 ng/mL, with a maximum value for CF3 sputum. Contrariwise, low MUC5B concentrations ranging from 0.388 (±0.038) to 3.95 (±0.061) ng/mL were measured for “low colonization” sputum samples. In line with literature data, very small amounts of MUC2 were found in all the CF sputum samples (0.271–2.56 ng/mL) with the only exception of CF2 (3.98 ± 0.148 ng/mL).

The transport of fluorescent hNPs through each CF sputum layer was assessed by the Transwell multi-plate assay.^29^ Trying to elucidate the role of the surface lipid, the behavior of PEI-containing hNPs engineered with either DPPC (PEI/siNFkB_DPPC_Rhod_) or DSPE-PEG (PEI/siNFkB_DSPE-PEG_Rhod,_) were compared. In fact, PEI did not play a crucial role on hNPs/mucus interactions, while it tuned siRNA entrapment, release, and subsequent transfection efficiency of the siRNA cargo. Results are reported in Figure 9 as the percentage of fluorescent hNPs permeated across the CF sputum layer and found in the SILF-containing donor compartment over time.

**Figure 8:**
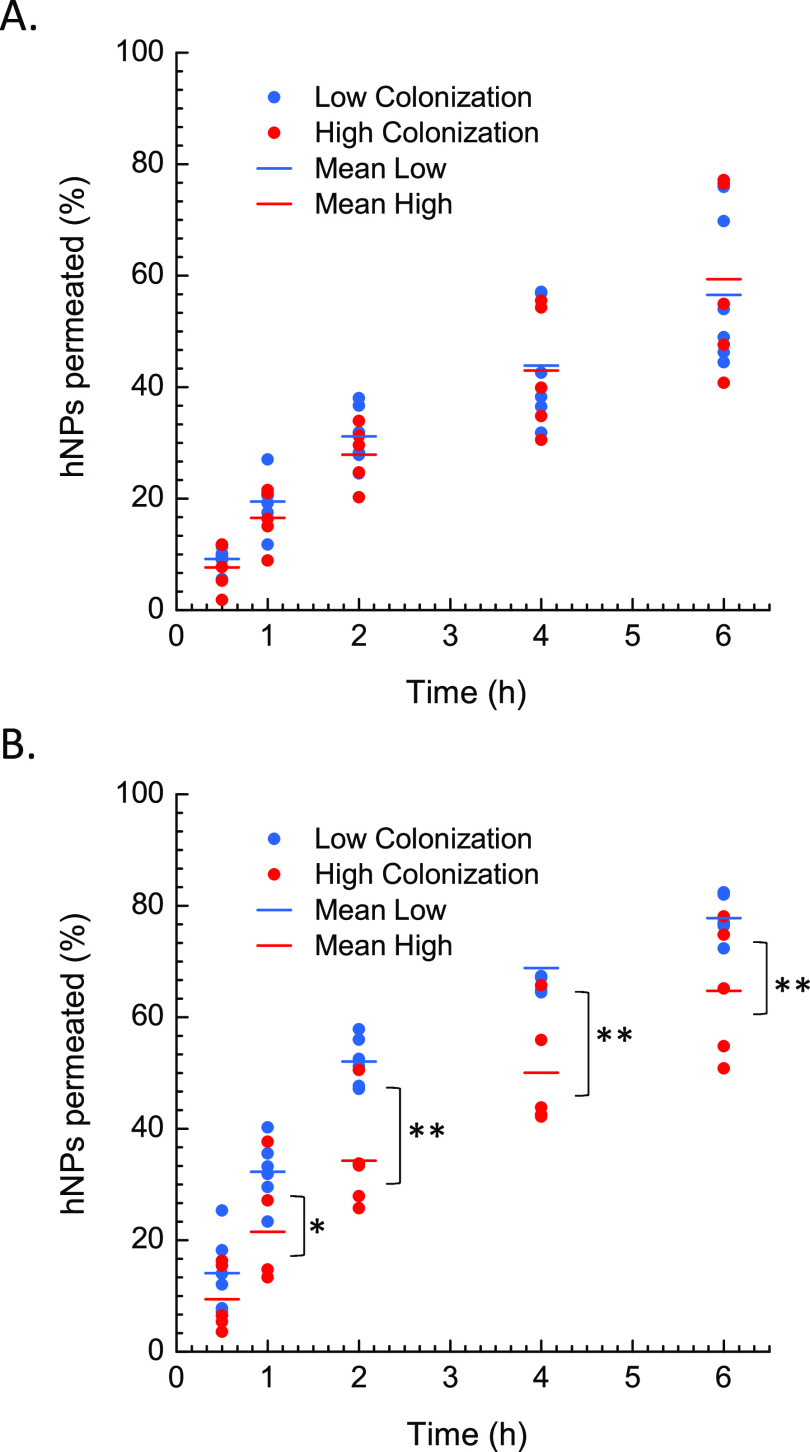
Percent amount of PEI/siNFkB_DPPC_Rhod_ (A) and PEI/siNFkB_DSPE-PEG_Rhod_ (B) permeated through different CF sputum samples over time. Data are mean values calculated on triplicate experiments performed in each CF sputum sample and are grouped according to the different number of microbial species isolated from the corresponding sample. Bars represent the mean percentage of hNPs permeated at each time point (*p < 0.1; **p < 0.05).

In each case, the diffusion profile varied with the tested sample (Figure 8). This trend can be likely attributed to the variable composition and viscosity of CF sputum samples (Figure 7), which are strongly affected by the health conditions of the corresponding CF donor.^48^ Noteworthy, the presence of PEG on the hNP surface significantly increased the permeation rate of the hNPs through poorly colonized (isolated microorganisms < 2) CF sputa (blue dots in Figure 8B). Nonetheless, a more complex sputum colonization is associated with a decreased positive effect of PEG on hNP permeation. In fact, the permeation rate of PEI/siNFkB_DSPE-PEG_Rhod_ in less complex CF sputa (blue dots in Figure 8B) was significantly higher than that of PEI/siNFkB_DPPC_Rhod_ (blue dots in Figure 8A) (*p* < 0.05 at time points 1, 2, 4, and 6 h). Contrariwise, no significant difference was observed in the permeation rate of PEI/siNFkB_DSPE-PEG_Rhod_ (blue dots in Figure 8B) as compared to PEI/siNFkB_DPPC_Rhod_ (blue dots in Figure 8A) (*p* ≫ 0.1 at each time point) in highly colonized CF sputum samples (i.e., high colonization group).

To confirm the ability of hNPs to assist the diffusion of the entrapped siRNA through CF sputum, studies were performed also with hNPs containing Alexa Fluor 647–labeled siRNA (siFluo) (Figure S6). The total amount of siFluo (free and encapsulated) found in the donor compartment at each time point confirmed similar transport kinetics. Nevertheless, except for the first time points (i.e., 30 min and 1 h time intervals), the presence of PEG on the hNP surface does not seem to enhance the permeation rate of siFluo through “low colonization” CF sputum samples (blue dots in Figure S6B).

As confirmed by *in vitro* release studies (Figure S7), the contribution of the amount of siRNA released in comparison to the total encapsulated siFluo over time should be considered.

The promoting effect of PEGylated hNPs on siRNA transport through CF mucus was even less apparent on the CF3 sample, which was not included in the overall data analysis, due to the peculiar behavior of siRNA-loaded hNPs in this sample (Figure S8). The sputum was obtained from a male 42 years-old patient infected with meticillin-resistant *Staphylococcus aureus*, *Aspergillus terreus*, *Achromobacter denitrificans*, *Comamonas acidovorans*, *Shingobacterium spiritivorum* (Table 1), some of the emerging pathogens in CF, and a very poor pulmonary function (FEV1 30%; FVC 57%). Increased sputum production is commonly associated with active fungal infections, as those of *Aspergillus spp*.^49^ Moreover, potential binding of conidial surface lectin of *Aspergillus spp*. to mucin glycans has been hypothesized, thus altering the mucus barrier properties.^50^ Meanwhile, aggregates consistent with biofilm were visualized in sputum samples from CF patients colonized with Achromobacter.^51^ Rheology measurements could not be performed due to the low volume of the CF sputum samples. Nevertheless, sample CF3 is macroscopically the most viscous one (Figure 7A). Furthermore, dsDNA and mucin analysis confirm the dominant role of MUC5B and MUC5AC in the barrier properties of this mucus toward the developed hNPs, partially contributed to by extracellular DNA (Figure 7B,C).

Overall, results point out a quite different and variable behavior of hNPs in CF sputa as compared to AM. Recent data suggest that commercial mucin from porcine stomach, type II, may present several structural alterations as compared to lab-purified MUC5AC, likely affecting mucin functionality in terms of gelation and adsorption.^52^ This could partly explain the different behavior of hNPs in AM, which is closer to that observed in “low colonization” sputa, characterized by low concentration of gel-forming mucins.

Nonetheless, different hNP interactions with the complex CF sputum matrices (Table 1, Figure 7) as compared to AM are feasible. This hypothesis was confirmed by assessing hNPs/CF sputum interactions by DLS (Figures S9 and S10). Though a slight increase of PEI/siNFkB_DPPC size, along with PDI, was observed in “low colonization” sputa (Figure S9A), the size distribution profile in “high colonization” samples was considerably more variable, with CF3 causing a manifest hNP aggregation (*D_H_* ≫ 1 μm; PDI ≈ 0.5) (Figure S9B). Of note, PEI/siNFkB_DSPE-PEG hNPs (Figure S10) displayed a very similar behavior to that of PEI/siNFkB_DPPC, suggesting some aggregation propensity of hNPs in “high colonization” CF samples, independently upon PEGylation.

### Nanoparticle Permeation in Mucus Covered Calu-3 Cell Monolayers

Upon diffusion in mucus, a successful siRNA delivery system must release the drug payload to the underlying cell layer, where the target is located. Thus, the ability of non-PEGylated and PEGylated hNPs to assist the siRNA transport through mucus-lined Calu-3 monolayers grown at ALI was further investigated. Calu-3 cells are widely used as a model for CF due to their high transepithelial resistance, mucus secretion, and expression of CFTR protein on the apical side when cultured on Transwell filters.^53,54^ When cultured at ALI, Calu-3 cells form a pseudostratified columnar epithelium secreting mucus, with a morphology very close to the airway epithelium in terms of the ultrastructure and secretory components, including the gel-forming mucin MUC5AC and electrical resistance.^55−57^

**Figure 9:**
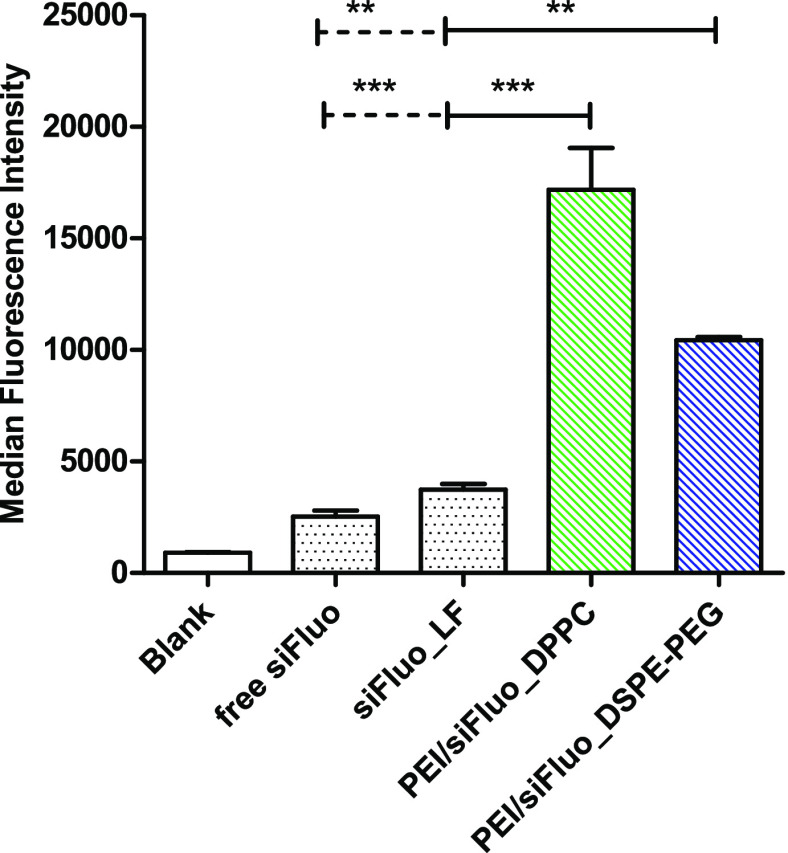
Cellular uptake in Calu-3 cells after transfection for 24 h with siFluo-loaded hNPs. As positive control, cells were transfected with siRNA/lipofectamine complexes (siFluo_LF). Free siRNA (free siFluo) was used as negative control. Data analysis was performed by GraphPad Prism 5.0 software. Results are reported as mean values ± SD. Significance was measured by one way ANOVA analysis (***p < 0.005; **p < 0.01).

Polarized epithelial Calu-3 cell layers (TEER value of at least 300 Ω*cm^2^) were first incubated with PEI/siFluo_DPPC and PEI/siFluo_DSPE-PEG for cell uptake studies. Based on the preliminary *in vitro* dose–response gene silencing studies (Figure S11), the experiments were performed at 20 nM siFluo. Both PEI-containing formulations show significant uptake in comparison to both free siRNA and siRNA/lipofectamine complexes. In particular, the best results are obtained with DPPC-engineered hNPs. (Figure 9).

**Figure 10:**
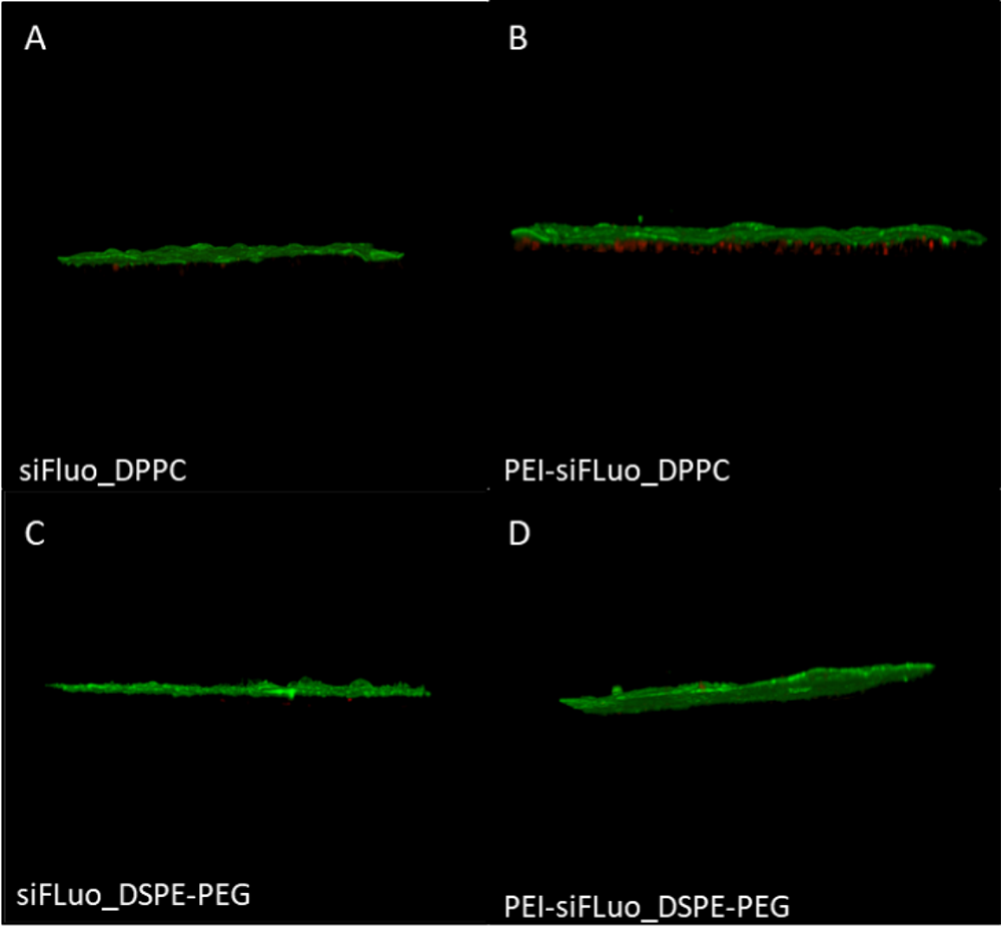
3D view created from confocal laser scanning microscopy sections of confluent Calu-3 monolayers grown at ALI exposed to siFluo_DPPC (A), PEI-siFluo_DPPC (B), siFluo_DSPE-PEG (C), or PEI-siFluo_DSPE-PEG (D) hNPs for 24 h and subsequent staining of mucus with AlexaFluor488-labeled wheat germ agglutinin. The green fluorescence corresponds to the mucus covering the cell surface, while red dots correspond to siRNA fluo loaded into the hNPs.

An initial ALI culture-based experiment was then completed to assess hNPs’ ability to deliver siRNA through the mucus covering the confluent Calu-3 monolayers (TEER values ≥ 300 Ω*cm^2^). The 3D view of the cell culture model is reported in Figure 10.

All the tested hNPs efficiently diffuse across the mucus layer covering the Calu-3 monolayer and were taken up intracellularly. Nevertheless, the Calu-3 uptake of siRNA-loaded PEI-DPPC hNPs appeared more efficient than that observed for the other hNP formulations.

These results are not surprising, since the mucus layer and the cell membrane are very complex and have different barriers, and thus particle engineering suitable to improve the permeation across the first may not be adequate for the second.^58^ This is the case for siRNA-loaded PEGylated hNPs, being able to enhance the diffusion of the particles through the CF sputum while limiting subsequent cell uptake.

### *In Vitro* Gene Silencing Activity

**Figure 11:**
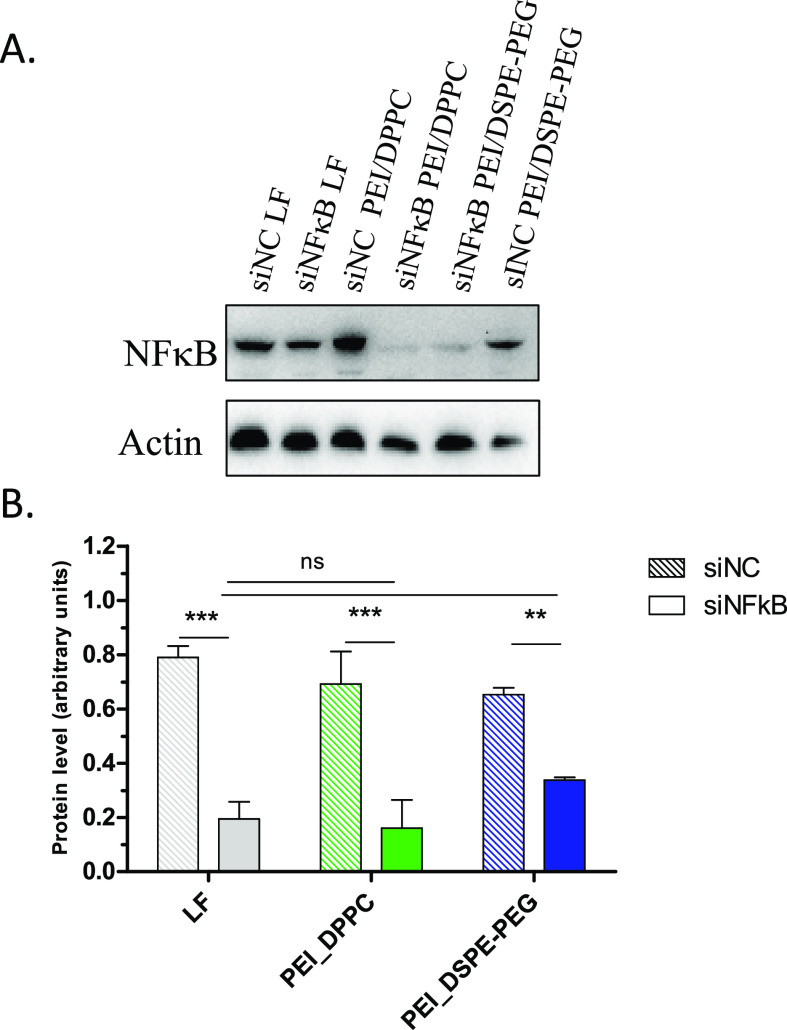
Representative Western blotting of protein extracts from 16HBE14o- cells. Before the transfection, the cells were stimulated for 4 h with LPS [25 μg/mL] to induce NFkB gene expression. The transfection was performed with 20 nM of siNFkB-loaded hNPs and siNC (siRNA non-coding) for 72 h. (A) The quantification of signals is shown. (B) The signal from cells treated with the siNFkB and lipofectamine complex is reported as positive control. Significance was measured by one way ANOVA analysis (***p < 0.005).

Once confirmed that hNPs are able to assist siRNA transport through mucus-covered lung epithelial cells, their capability to release the siRNA cargo in the cytosol and to knockdown NFκB gene expression was investigated in LPS-stimulated 16HBE14o- cells. Stimulation of cells with LPS from *E. coli* caused an increase of NFκB expression, which was efficiently inhibited by 20 nM siNFκB (Figure S11). *In vitro* knockdown experiments were performed at the corresponding concentration of siNFκB-loaded hNPs. The preliminary MTT assay confirmed that hNPs do not considerably affect 16HBE14o- cell viability at the tested concentrations (Figure S12). Despite the relatively low amount of siNFκB released from hNPs in buffer over 72 h (Figure S7), both PEGylated and non-PEGylated hNPs displayed a silencing effect comparable to that observed with lipofectamine, the reference transfection reagent (Figure 11). This observation is more evident for PEI/siNFκB_DPPC hNPs than for PEI/siNFκB_DSPE-PEG hNPs, likely due to the faster siNFkB release. Thus, results confirm the ability of the developed hNPs to release siNFkB intracellularly and to induce *in vitro* inhibition of NFκB gene overexpression, likely preventing progressive and fatal degradation inside intracellular organelles (i.e., endosomes).

## Conclusions

One of the main challenges in the field of siRNA delivery to the lungs remains the development of strategies able to efficiently assist the transport of the macromolecular cargo across the mucus-covered airway epithelium. Here, non-PEGylated and PEGylated siRNA-loaded lipid/PLGA polymer hybrid nanoparticles, that is, hNPs, were successfully developed and proposed to tackle this barrier. Valuable information on factors governing hNP interactions with mucus, its main components, and lung epithelial cells were achieved combining different techniques and model systems. We found that PEGylation does not make the difference when the mucus barrier properties are dominated by pathology-associated proteins, such as the gel-forming mucins MUC5AC and MUC5B (i.e., sputum from cystic fibrosis patients). Nevertheless, subsequent uptake of hNPs in mucus-covered human airway epithelial cells grown at the air–liquid interface can be prevented by PEGylation. Thus, the presence of a biomimetic lipid shell is enough to assist hNP permeation across mucus, but it becomes essential to achieve both mucus penetration and cellular internalization as demonstrated also through *in vitro* gene silencing data. Overall, results highlight how a thorough understanding of nanoparticle behaviors in the physiological lung environment is the key for designing a delivery system allowing an efficient translocation of siRNA to its cell target in the lung. Data confirm the potential of hNPs as carriers for pulmonary delivery of siNFκB for local treatment of CF lung disease and prompt toward in depth investigation of their therapeutic effectiveness *in vitro* and *in vivo*.

## Supporting Information Available

(Figure S1) SAXS spectra of 1% w/v water dispersions of siNFkB_DSPE-PEG and PEI/siNFkB_DSPE-PEG hNPs; (Figure S2) evaluation of siRNA-PEI complexation through electrophoresis; (Figure S3) FALT measurements of the polymer shell thickness of siNFkB-loaded hNPs; (Figure S4) ^1^H NMR spectra displaying the residual PEG signal at 3.62 ppm; (Figure S5) decay of the PEG peak intensity at increasing applied gradient in DOSY - LEDBPGP2s experiments; (Figure S6) percent amount of siFluo transported over time through different CF sputum samples; (Figure S7) *in vitro* release kinetics of siNFkB from PEI/siNFkB_DPPC and PEI/siNFkB_DSPE-PEG hNPs; (Figure S8) percent amount of PEI/siNFkB_DPPC_Rhod_ and PEI/siNFkB_DSPE-PEG_Rhod_ permeated through the CF3 sputum sample over time; (Figures S9 and S10) *in vitro* assessment of PEI/siNFkB_DPPC_Rhod_ and PEI/siNFkB_DSPE-PEG_Rhod_ interactions with CF sputa; (Figure S11) *in vitro* gene-silencing effect of different concentrations of siNFkB on LPS-stimulated 16HBE14o- cell; and (Figure S12) cytotoxicity of hNPs on 16HBE14o- cells via the MTT assay (PDF)
