# Supplementary material for: Hybrid Lipid/Polymer Nanoparticles to Tackle the Cystic Fibrosis Mucus Barrier in siRNA Delivery to the Lungs: Does PEGylation Make the Difference?
Source: ACS Appl Mater Interfaces. 2022 Feb 2;14(6):7565–78. doi: 10.1021/acsami.1c14975 (PMC8855343; doi:10.1021/acsami.1c14975)
Supplement: Supplementary file 1 — am1c14975_si_001.pdf [file am1c14975_si_001.pdf]

SUPPORTING INFORMATION

Hybrid lipid/polymer nanoparticles to tackle the cystic fibrosis mucus barrier in siRNA delivery to the lungs: does PEGylation make the difference?

Gemma Conte[1, ‡], Gabriella Costabile[2, ‡], Domizia Baldassi[3], Valeria Rondelli[4], Rosaria Bassi[4], Diego Colombo[4], Giulia Linardos[5], Ersilia V. Fiscarelli[5], Raffaella Sorrentino[6], Agnese Miro[2], Fabiana Quaglia[2], Paola Brocca[4], Ivana d'Angelo[1 *], Olivia M. Merkel[3], Francesca Ungaro[2 *]

1. Di.S.T.A.Bi.F., University of Campania Luigi Vanvitelli, 81100 Caserta, Italy.
2. Department of Pharmacy, University of Napoli Federico II, 80131, Napoli, Italy
3. Department of Pharmacy, Pharmaceutical Technology and Biopharmacy, Ludwig-Maximilians-Universität, München, 81377, Munich, Germany.
4. Department of Medical Biotechnologies and Translational Medicine, University of Milano, 20090, Segrate (MI), Italy
5. Children's Hospital Bambino Gesù IRCCS, Rome, Italy.
6. Department of Molecular Medicine and Medical Biotechnologies, University of Napoli Federico II, 80131, Napoli, Italy.

*Corresponding Authors

* Ivana d'Angelo, Di.S.T.A.Bi.F., University of Campania Luigi Vanvitelli, Via Vivaldi 43, 81100 Caserta, Italy. ORCID: orcid.org/0000-0003-0444-0444. E-mail: ivana.dangelo@unicampania.it

* Francesca Ungaro, Department of Pharmacy, University of Napoli Federico II, Via D Montesano 49, 80131 Napoli, Italy. ORCID: orcid.org/0000-0003-0850-9533. E-mail: ungaro@unina.it

‡These authors contributed equally

SUPPORTING INFORMATION

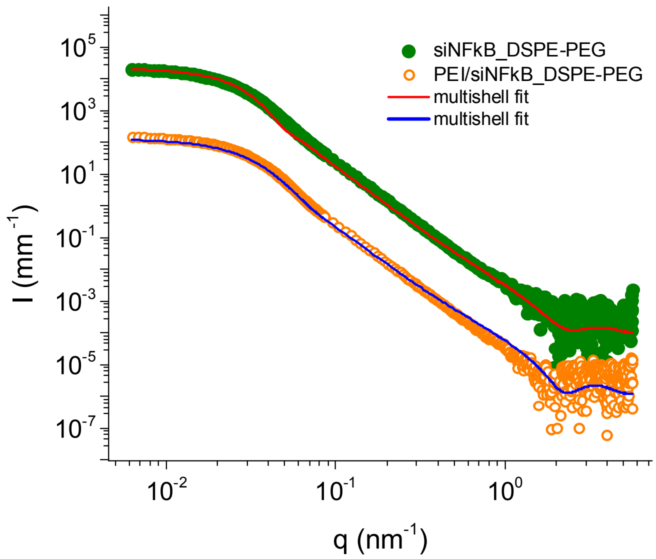

Figure S1. SAXS spectra of 1% w/v water dispersion of siNFkB_DSPE-PEG (multiplied by 100) and PEI/siNFkB_DSPE-PEG hNPs with their fit by a core-multishell form factor. The phospholipid monolayer is built by a 1.7 nm lipid shell and a 0.7 nm headgroup shell for of siNFkB_DSPE-PEG and by a 1.8 nm lipid shell and a 0.7 nm headgroup shell for PEI/siNFkB_DSPE-PEG hNPs. Fit was performed applying spherical core-multishells form factor using the routines developed in the SaSView program (SasView - Small Angle Scattering Analysis, 2019. Available at: https://www.sasview.org/).

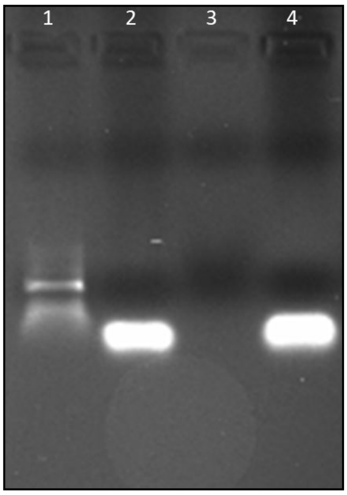

Figure S2. Agarose gel electrophoresis of siNFκB/PEI complex. Lane 1: Low range single strand RNA ladder, Lane 2: siRNA free 0.2 nmol, Lane 3: siRNA/PEI complex prepared from 0.2 nmol siRNA and 3.2 μg PEI, Lane 4: siRNA/PEI complex prepared from 0.2 nmol siRNA and 3.2 μg PEI upon incubation with heparin as control. The absence of the band in Lane 3 demonstrates the successful complexation of siRNA/PEI, also validated in Lane 4 when the heparin replaces PEI in the formation of the complex.

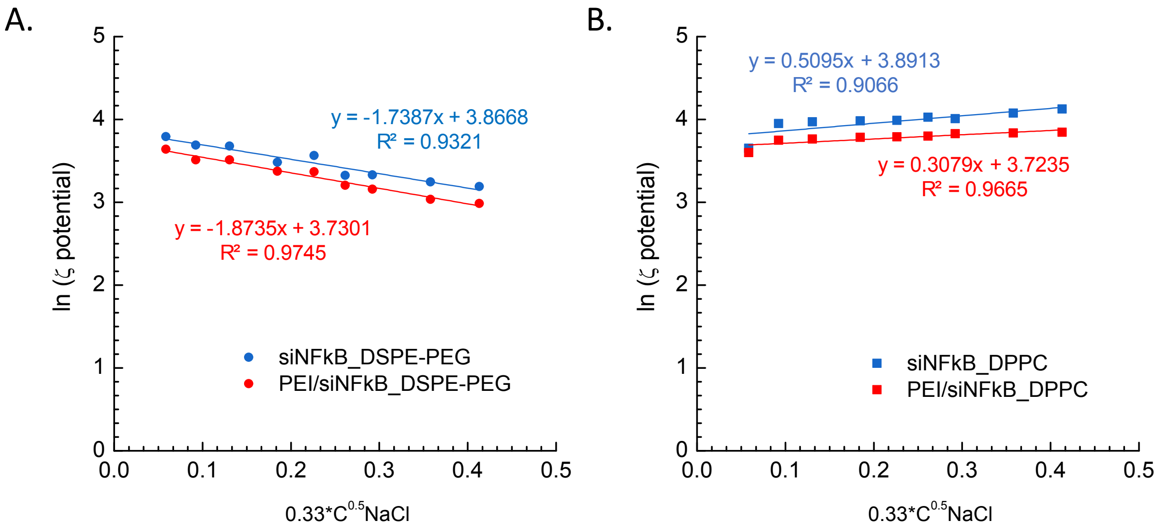

Figure S3. FALT measurements of the polymer shell thickness of siNFkB-loaded DSPE-PEG/PLGA hNPs (A) and DPPC/PLGA hNPs (B). Plots of ln(ζ) against k (k =3.3 $C^{0.5}$, where k^{-1} is the Debye length and C is the concentration of NaCl in the dispersion) show the thickness of the polymer layer (nm) as the slope of linear regression Data are mean values of triplicate analysis performed on 3 different batches.

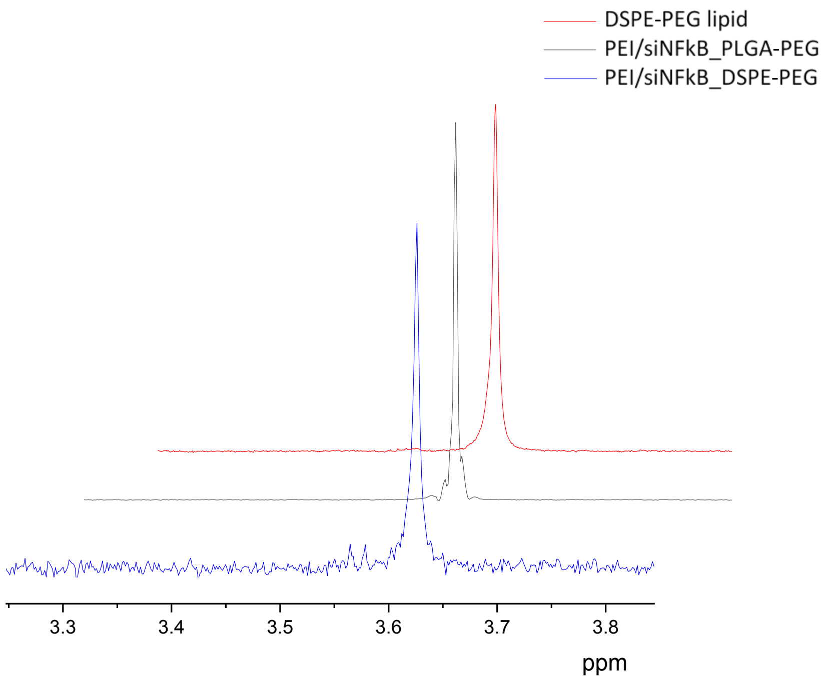

Figure S4. Residual PEG signal at 3.62 ppm in PEI/siNFkB_DSPE-PEG hNPs (bottom), PEI/siNFkB_PLGA-PEG nanoparticles (center) and DSPE-PEG lipid x 0.13 (top) shifted and rescaled for the sake of comparison. Spectra were obtained by a spin echo sequence to remove residual ethanol signals in the spectrum.

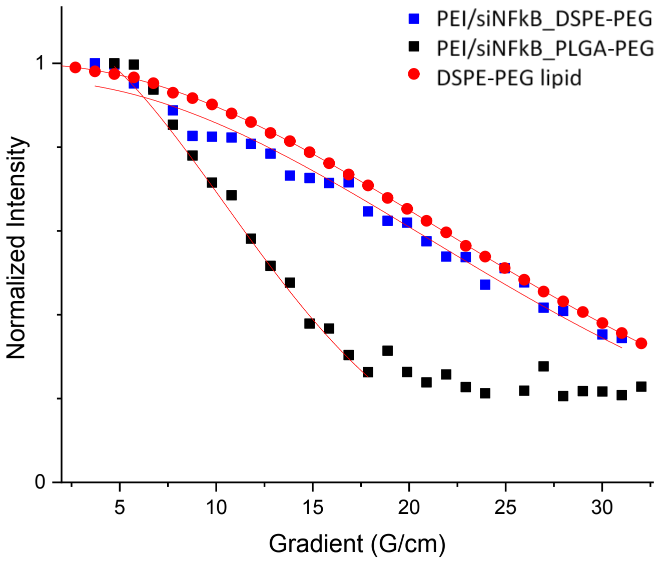

Figure S5. Decay of the PEG peak intensity at increasing applied gradient in DOSY - LEDBPGP2s experiments. The decay of the high-resolution signal in PEI/siNFkB_DSPE-PEG (red) is similar to that from DSPE-PEG micelles (blue) while PEI/siNFkB_PLGA-PEG nanoparticle dispersion shows faster moving PEG components for the same experimental conditions. The measurements were performed applying magnetic field gradient power in the limit of linearity that prevented to reach a complete cancellation of the PEG signal at the highest gradient, but a decrease over of the order of e−1 was always obtained. The noise in the experimental points are due to the low intensity of the residual PEG peak.

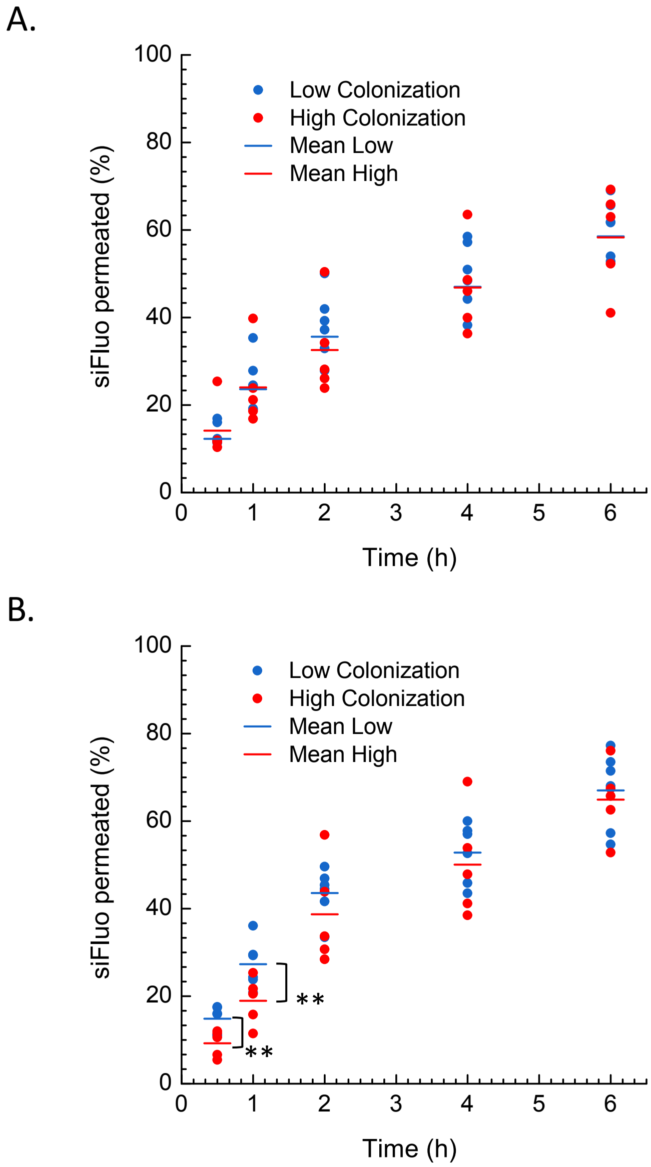

Figure S6. Percent amount of siFluo transported over time by PEI/siFluo_DPPC (A) and PEI/siFluo_DSPE-PEG (B) through different CF sputum samples. Data are mean values calculated on triplicate experiments performed in each CF sputum sample and are grouped according to the different number of microbial species isolated from the corresponding sample. Bars represent the mean percentage of siFluo permeated at each time point (** $p<0.05$).

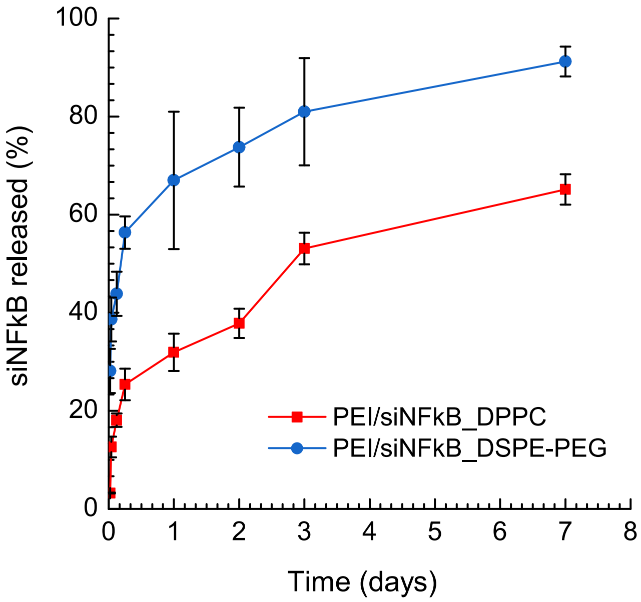

Figure S7. In vitro release kinetics of siRNA from PEI/siNFkB_DPPC and PEI/siNFkB_DSPE-PEG in PBS at pH 7.2 and 37°C. Data are mean values calculated on triplicate experiments ± standard deviation (SD).

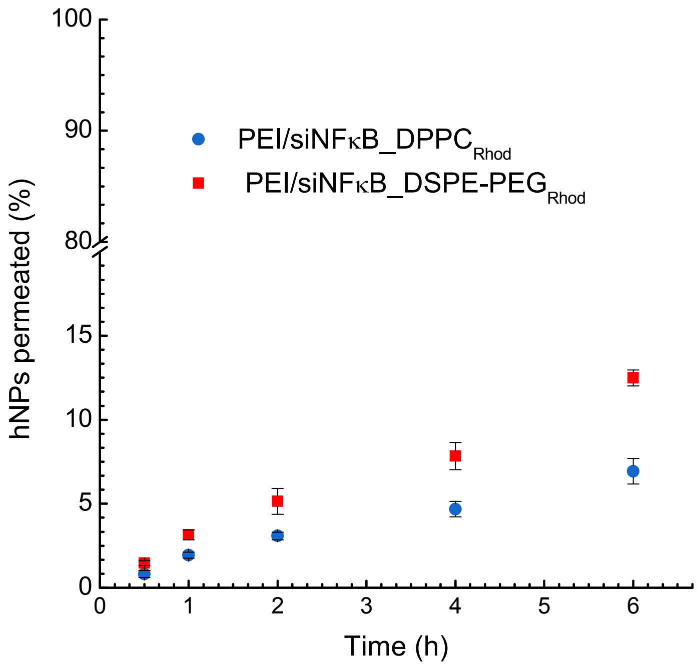

Figure S8. Percent amount of PEI/siNFkB_DPPC$_{Rhod}$ and PEI/siNFkB_DSPE-PEG$_{Rhod}$ permeated through CF3 sputum sample over time. Data are mean values calculated on triplicate experiments ± standard deviation (SD).

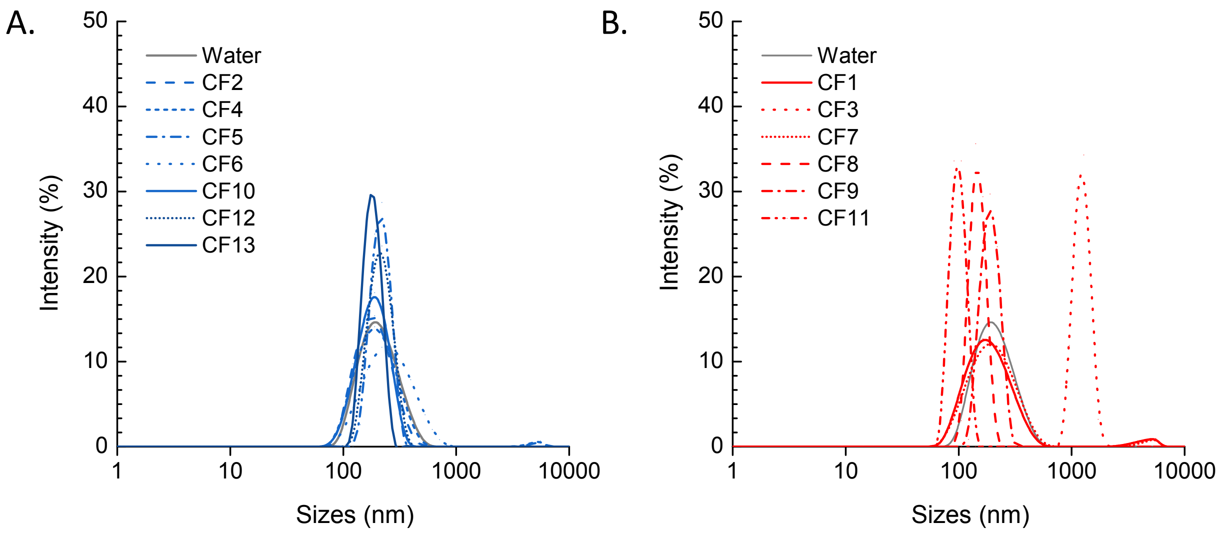

	Size (nm)	PDI
**Water**	161.3 ± 3.8	0.170 ± 0.04
**CF2**	197.3 ± 3.7	0.444 ± 0.07
**CF4**	220.6 ± 1.0	0.367 ± 0.53
**CF5**	229.4 ± 6.7	0.371 ± 0.44
**CF6**	257.9 ± 2.2	0.345 ± 0.74
**CF10**	184.6 ± 1.7	0.371 ± 0.03
**CF12**	227.3 ± 2.4	0.448 ± 0.04
**CF13**	197.2 ± 1.2	0.413 ± 0.09

	Size (nm)	PDI
**Water**	161.3 ± 3.8	0.170 ± 0.04
**CF1**	190.4 ± 1.04	0.361 ± 0.01
**CF3**	1280 ± 1.41	0.471 ± 0.43
**CF7**	193.6 ± 2.10	0.279 ± 0.09
**CF8**	142.6 ± 1.91	0.271 ± 0.04
**CF9**	190.0 ± 1.42	0.413 ± 0.06
**CF11**	106.4 ± 2.66	0.106 ± 0.04

Figure S9. *In vitro* assessment of PEI/siNFkB_DPPC hNPs interactions with CF sputa. Representative size distribution by intensity profile and corresponding D_H and PDI values of hNPs in the presence of "low colonization" (A) and "high colonization" (B) CF sputa. The size distribution profile and corresponding size/PDI of hNP dispersions in water are reported as controls.

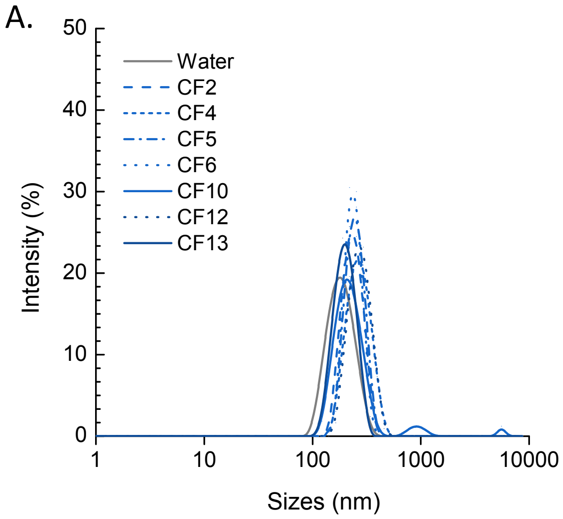

	Size (nm)	PDI
**Water**	165.5 ± 14.1	0.105 ± 0.069
**CF2**	221.3 ± 1.4	0.211 ± 0.211
**CF4**	254.3 ± 2.2	0.441 ± 0.097
**CF5**	279.3 ± 3.1	0.336 ± 0.227
**CF6**	286.4 ± 2.1	0.412 ± 0.194
**CF10**	224.1 ± 1.1	0.410 ± 0.074
**CF12**	259.1 ± 2.6	0.243 ± 0.147
**CF13**	193.4 ± 4.6	0.164 ± 0.077

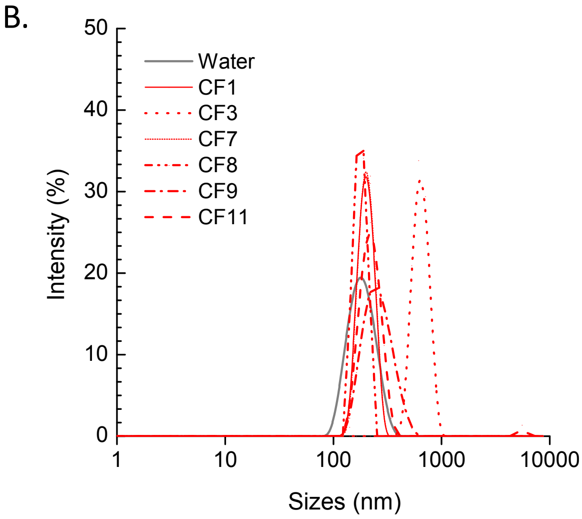

	Size (nm)	PDI
**Water**	165.5 ± 14.1	0.105 ± 0.069
**CF1**	194.1 ± 1.24	0.257 ± 0.064
**CF3**	615.6 ± 3.47	0.669 ± 0.117
**CF7**	247.9 ± 4.65	0.665 ± 0.070
**CF8**	197.6 ± 2.44	0.210 ± 0.022
**CF9**	245.6 ± 3.16	0.141 ± 0.069
**CF11**	217.6 ± 1.66	0.341 ± 0.120

Figure S10. *In vitro* assessment of PEI/siNFkB_DSPE-PEG hNPs interactions with CF sputa. Representative size distribution by intensity profile and corresponding D_H and PDI values of hNPs in the presence of "low colonization" (A) and "high colonization" (B) CF sputa. The size distribution profile and corresponding size/PDI of hNP dispersions in water are reported as controls.

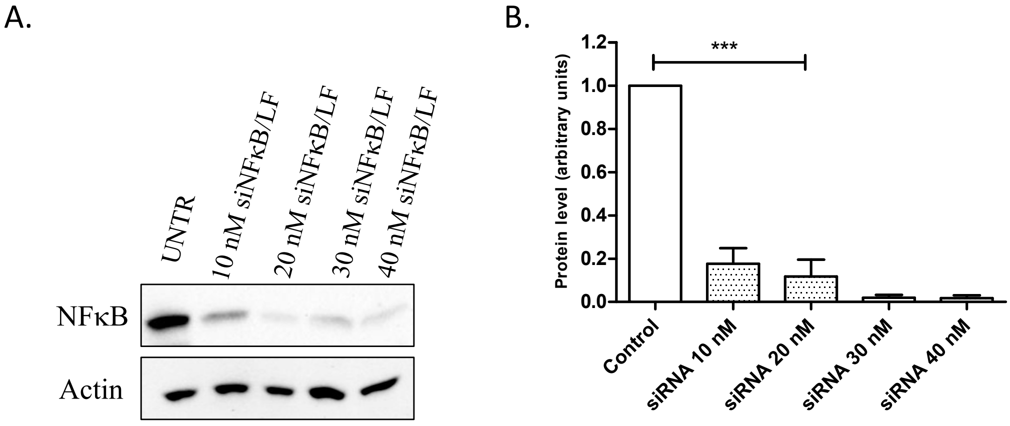

Figure S11. *In vitro* gene-silencing effect of different concentrations of siNFkB on LPS-stimulated 16HBE14o- cell. Before the transfection the cells were stimulated for 4h with LPS [25µg/mL] to induce NFkB gene expression. Representative Western blotting analysis of protein extracts from 16HBE14o- cell at 72h treated with different amounts of siNFκB/lipofectamine complexes (A). The quantification of signals is shown (B). The signal of untreated cells (Control) was set as 1, and the treated samples were normalized accordingly

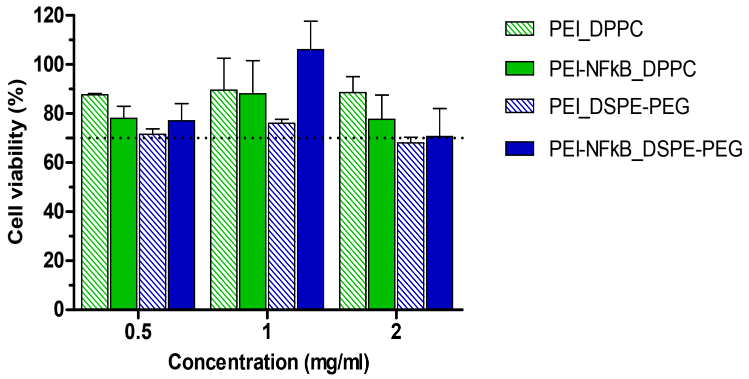

Figure S12. Cytotoxicity of hNPs on 16HBE14o- cells via MTT assay. 16HBE14o- cells were exposed to increasing concentrations of PEI/NFkB_DPPC and PEI/NFkB_DSPE-PEG for 24h. Unloaded hNPs were used as controls, Results are expressed as cell viability percentage (mean ± SEM) (n=3).
